# Cyanobacteria-derived near-infrared autofluorescent exosomes enabling synergistic brain lesion imaging and neuroprotection

**DOI:** 10.1016/j.bioactmat.2026.04.027

**Published:** 2026-04-20

**Authors:** Jingmei Pan, Yayun Wang, Yikun Feng, Jiaoyang Wang, Qiongya Huang, Lei Yan, Xiaobo Zhou, Huili Sun, Huaiyu Wang, Qu Wei

**Affiliations:** a Institute of Biomedical Engineering, College of Medicine, Southwest Jiaotong University, Chengdu, 610031, China; b Center for AI-Driven Medical Research, Shenzhen Institute of Advanced Technology, Chinese Academy of Sciences, Shenzhen, 518055, China; c 3Department of Basic Medicine, School of Medicine, Taizhou University, Taizhou, 318000, China; d Center for Computational Systems Medicine, School of Biomedical Informatics, The University of Texas Health Science Center at Houston, Houston, TX, USA; e School of Life Sciences and Engineering, Southwest Jiaotong University, Chengdu, 610031, China; f West China Biomedical Big Data Center, West China Hospital, Sichuan University, Chengdu, 610041, China

**Keywords:** Exosomes, Neuroprotection, Ischemic stroke, Blood-brain barrier penetration, Stroke theranostics

## Abstract

Precision diagnosis and treatment of central nervous system (CNS) diseases are hindered by limited probe penetration, toxicity risks, and low imaging signal-to-noise ratio (SNR). The blood-brain barrier (BBB) further restricts drug delivery, especially in stroke therapy. This study proposes and validates a natural exosome (sExos) from cyanobacteria, featuring intrinsic near-infrared-I (NIR-I) autofluorescence, with strong imaging and neuroprotective functions. As a theranostic nanoplatform, sExos enable integrated diagnosis and treatment of stroke and other brain disorders. Enriched with the fluorescent phycobiliprotein ApcE, sExos support label-free, high-SNR brain imaging in the NIR-I window. *In vivo*, sExos cross the BBB and accumulate in ischemic lesions, enabling dynamic visualization. Mechanistically, sExos regulate lipid metabolism and inhibit NF-κB signaling, reducing oxidative stress and neuroinflammation, while promoting neural recovery. Toxicity and immunogenicity evaluations confirm excellent biocompatibility and safety. In summary, this naturally autofluorescent exosome offers a label-free, brain-penetrant, and therapeutically promising imaging-intervention strategy, opening avenues for noninvasive stroke therapy and precision CNS disease management.

## Highlights

• A cyanobacteria-derived, naturally autofluorescent exosome is developed for BBB-penetrant CNS theranostics.
• Intrinsic NIR-I autofluorescence enables label-free, high-SNR brain imaging *in vivo.*
• The exosomes cross the BBB and selectively accumulate in ischemic brain lesions.
• Neuroprotection is achieved *via* lipid metabolism regulation and NF-κB inhibition.
• Favorable biocompatibility supports translational potential.

## Graphical abstract

**Figure:**
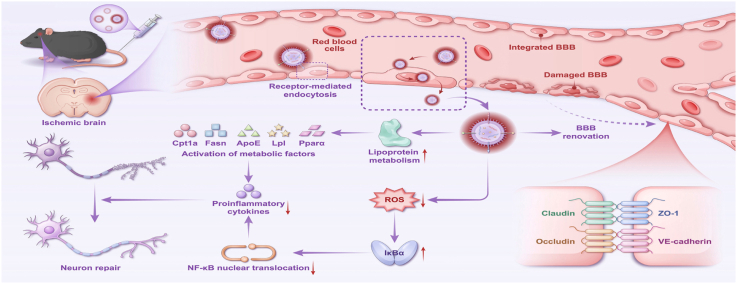

## 1 Introduction

CNS disorders, including stroke, neurodegenerative diseases, and traumatic brain injury, represent a major global health burden due to their high incidence, disability rates, and limited therapeutic efficacy [[1], [2], [3]]. Achieving precise diagnosis and targeted treatment is critical for improving clinical outcomes. However, current diagnostic and therapeutic strategies remain severely constrained by the BBB, which hinders the effective delivery of therapeutic agents and imaging probes [[4], [5], [6], [7], [8]]. Moreover, conventional imaging or therapeutic agents often suffer from poor biocompatibility, low SNR, and potential systemic toxicity [[9], [10], [11], [12], [13], [14]].

In CNS precision medicine, overcoming the BBB to achieve efficient targeting and real-time monitoring remains a key scientific challenge. Exosomes, particularly those derived from mammalian sources such as mesenchymal stem cells, have emerged as promising nanocarriers owing to their intrinsic biocompatibility, nanoscale size, native lipid bilayer membrane, and capacity to carry functional biomolecules [[15], [16], [17]]. These physicochemical and biological features endow exosomes with the potential to traverse the BBB *via* membrane protein–mediated transcytosis and cellular uptake pathways [18]; meanwhile, the endogenous enrichment of functional nucleic acids and proteins enables exosomes to exert intrinsic bioregulatory and therapeutic effects even in the absence of exogenous drug loading, highlighting their unique “carrier–therapeutic” integrated nature [19,20]. Nonetheless, challenges including limited scalability, high production costs, and ethical concerns significantly hinder the clinical translation of cell-derived exosomes [21]. Moreover, current exosome-based platforms often suffer from insufficient imaging sensitivity, limited *in vivo* visualization capability, and suboptimal integration of diagnostic and therapeutic functions, thereby leaving substantial room for further optimization and motivating the development of next-generation functionalized exosome systems.

In this study, we propose an intelligent strategy by employing natural exosomes derived from the photosynthetic cyanobacterium *Synechococcus elongatus* PCC 7942 (SYN) as a novel CNS-targeted theranostic nanoplatform. This photosynthetic microorganism offers inherent advantages, including rapid proliferation, ease of large-scale culture, and low immunogenicity [[22], [23], [24], [25], [26], [27], [28]]. Consequently, the SYN-derived exosomes (sExos) hold great potential in CNS treatment, which have not been deeply explored yet. Notably, we report for the first time that sExos possess intrinsic NIR-I autofluorescence, a previously undocumented optical feature that enables high-SNR *in vivo* imaging without external labeling. Combined with their nanoscale dimensions and excellent biocompatibility, sExos present an ideal dual-function platform integrating non-invasive imaging with neuroprotective capabilities.

**Fig. 1:**
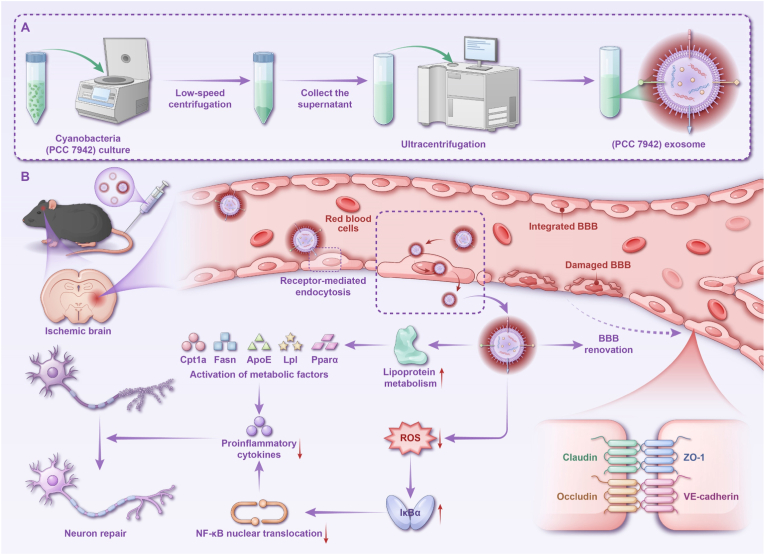
Schematic illustration of the extraction, characterization, and mechanism of action of sExos in stroke therapy. (A) Extraction and purification of sExos from SYN. (B) Mechanisms of cyanobacterial exosome-mediated stroke therapy. After injection, the intrinsically fluorescent sExos cross the BBB *via* receptor-mediated endocytosis, allowing them to enter the ischemic brain. They modulate lipid metabolism by regulating ApoE, Lpl, and Pparα, facilitating neuronal repair. Additionally, sExos suppress pro-inflammatory cytokines, reduce ROS, inhibit NF-κB signaling, and alleviate neuroinflammation, ultimately reducing neuronal damage and contributing to BBB renovation and enhancing recovery in stroke models.

Building on these findings, we developed a multifunctional nanoplatform that integrates NIR imaging and therapeutic functions for the precise diagnosis and treatment of ischemic stroke, as illustrated in (Fig. 1A and B). In a murine stroke model, sExos demonstrated effective BBB penetration and selectively accumulated in ischemic regions, enabling real-time, label-free dynamic visualization of cerebral infarcts. Mechanistically, sExos modulated lipid metabolism and inhibited NF-κB pathway activation, thereby reducing oxidative stress and neuroinflammation while promoting neuronal survival and functional recovery. Comprehensive toxicological and immunological evaluations confirmed excellent biosafety, supporting the development of a scalable, low-toxicity, functionally integrated platform for precision diagnosis and therapy of neurological diseases.

## 2 Materials and methods

### 2.1 Cultivation, collection, extraction and purification of exosomes secreted by PCC 7942

The *Synechococcus elongatus* PCC 7942 strain was obtained from the Freshwater Algae Culture Collection of the Institute of Hydrobiology, Chinese Academy of Sciences (FACHB-1061, Wuhan, China). The strain was cultured in BG-11 media under continuous shaking at 30 °C and 125 rpm. Illumination was provided by white fluorescent light at an intensity of 2500-3500 lx with a 12 h light/12 h dark photoperiod. Cyanobacteria were collected by centrifugation at 2900×*g* for 5 min, and the supernatant was discarded. The cyanobacteria pellet was washed three times with phosphate-buffered saline (PBS) to obtain cyanobacteria clusters for subsequent experiments.

sExos secreted by PCC 7942 were extracted *via* differential centrifugation. When the optical density (OD) of the culture reached 0.8-1.0, the cyanobacterial cells and debris were removed by centrifugation at 7000×*g* for 15 min at 4 °C. The supernatant was then filtered through a 0.45 μm polyvinylidene fluoride (PVDF) membrane, followed by ultracentrifugation at 200,000×*g* for 1.5 h to eliminate impurities further. Finally, the purified sExo suspension was filtered through a 0.22 μm membrane to prevent potential contamination. The resulting sExo samples were stored in liquid nitrogen for subsequent use.

### 2.2 Cell strain and cultivation conditions

Rat PC-12 neuronal cells, human SH-SY5Y neuronal cells, and mouse bEnd.3 brain microvascular endothelial cells were obtained from the Cell Bank of the Chinese Academy of Sciences. bEnd.3 cells were cultured in Dulbecco's Modified Eagle's Medium (HyClone) supplemented with 10% fetal bovine serum (FBS). PC-12 cells were maintained in RPMI 1640 medium (HyClone) with 10% FBS, whereas SH-SY5Y cells were cultured in a 1:1 mixture of DMEM and F12 medium (HyClone) supplemented with 10% FBS and 1% non-essential amino acids to support neuronal differentiation and viability. All cell types were incubated at 37 °C in a humidified atmosphere containing 5% CO_2_.

### 2.3 Experimental animals

Specific pathogen-free (SPF) C57BL/6J male mice (25-30 g) were purchased from Beijing Huafukang Biotechnology Co., Ltd. The mice were aged 8 to 12 weeks and maintained in an environment with a temperature of 25 °C and a humidity of 55%. All animal experiments were approved by the Ethics Committee for Animal Research, Institutional Animal Care and Use Committee of Southwest Jiaotong University (Approval number: SWJTU-2403-NSFC(079)).

### 2.4 Proteomic sequencing analysis

For proteomics sequencing analysis of cyanobacterial cells and cyanobacterial extracellular vesicles, proteins were first extracted from the samples by adding an appropriate amount of protein lysis buffer (8 M urea +1% SDS, containing a protease inhibitor). The samples were subjected to three cycles of sonication using a high-throughput tissue grinder, each lasting 40 s, followed by 30 min of lysis on ice with vortexing for 5-10 s every 5 min. The samples were then centrifuged at 12,000×*g* for 30 min at 4 °C, and the supernatant was collected for protein quantification *via* the BCA method. After quantification, preliminary analysis was performed *via* SDS-PAGE. Next, 100 μg protein samples were treated with triethylammonium bicarbonate (TEAB) buffer for reduction and alkylation, followed by precipitation with precooled acetone. After precipitation, the samples were dissolved in TEAB, and trypsin was added at a 1:50 enzyme-to-protein mass ratio for overnight digestion at 37 °C. The digested peptides were vacuum-dried, then redissolved in 0.1% trifluoroacetic acid (TFA), desalted using HLB columns, and finally concentrated using a vacuum concentrator. Peptide quantification was performed *via* a Thermo Fisher Scientific quantification kit. Peptide separation was performed using a Vanquish Neo chromatograph, and mass spectrometry analysis was conducted with an Orbitrap Astral mass spectrometer in DIA mode, with a scan range of 100-1700 *m*/*z*. The raw DIA data were imported into Spectronaut™ 18 software for database search analysis, with parameters set to protein FDR ≤0.01, peptide FDR ≤0.01, and peptide confidence ≥99%, excluding shared and modified peptides. Finally, all the data were uploaded to the Megji Cloud platform, and differential analysis between groups was performed using the *t*-test function in R. Significantly different proteins (*P* < 0.05 and fold change >1.2) were identified. Gene Ontology (GO) annotation and KEGG pathway analysis were conducted for the differentially expressed proteins, and protein-protein interaction networks were analyzed *via* String v11.5 software.

### 2.5 Protein-protein docking methodology

Due to the lack of fully resolved experimental structures for several candidate proteins, the three-dimensional structures of ICAM-1, α_4_β_1_, and P-glycoprotein were predicted from their amino acid sequences using AlphaFold3. Specifically, α_4_β_1_ was modeled as a heterodimeric complex composed of the α_4_ and β_1_ subunits. Structural information for candidate proteins was preferentially retrieved from the UniProt database. For proteins with missing or incomplete structural data, AlphaFold3 was employed to generate high-confidence structural predictions and complement the unavailable regions.

Subsequently, protein–protein docking was performed using the HDOCK server under default parameters, employing a global blind docking strategy without predefined binding sites. The resulting docking conformations were ranked according to the docking score, and the top three predicted complexes were selected for further interaction analysis. Structural visualization and analysis of the docked complexes were conducted using PyMOL.

### 2.6 Endocytic pathway analysis of sExo transcytosis across the BBB

To systematically elucidate the endocytic mechanisms underlying sExo transport across the BBB, a series of inhibitor-based assays targeting distinct uptake pathways was performed. Briefly, bEnd.3 cells were seeded onto Transwell inserts and cultured until a confluent monolayer was established. Cells were then pretreated with specific endocytic inhibitors, including chlorpromazine (CPZ, 15 μM) for clathrin-mediated endocytosis inhibition, genistein (200 μM) for caveolae-mediated endocytosis inhibition, and EIPA (10 μM) for macropinocytosis inhibition, at 37 °C for 30 min. Following pretreatment, labeled sExos were added to the apical chamber and incubated at 37 °C for 1 h. In parallel, a 4 °C incubation group was included to assess the energy dependence of the uptake process. After incubation, the medium in the basolateral chamber was collected, and the translocation efficiency of sExos was quantitatively evaluated by measuring fluorescence intensity.

### 2.7 Characterization of cyanobacteria and sExos

The morphology and size of the cyanobacteria and sExos were analyzed *via* transmission electron microscopy (TEM, JEOL Ltd., Japan) and scanning electron microscopy. The hydrodynamic diameter and ζ-potential of the exosomes were measured using dynamic light scattering (DLS, ZETA-SIZER, MALVERN NanoZS90, Malvern Ltd., UK).

### 2.8 Biosafety evaluation

Cell compatibility was evaluated *via* the Alamar blue assay and the live/dead staining method. For the Alamar Blue assay, PC-12 cells and bEnd.3 cells were seeded in 48-well plates (5 × 10^4^ cells/well) and cultured for 24 h sExos at various concentrations (25-300 μg/mL) were then added to each well. After 24 h, the culture medium was removed, and 300 μL of a mixture containing 10% Alamar Blue, 80% medium 199 (Gibco), and 10% fetal bovine serum (v/v) was added for an additional 3 h of incubation. Cell viability was assessed by measuring the absorbance *via* a microplate reader (Multiskan FC, Thermo Fisher). For the live/dead staining assay, cells were cocultured with sExos at various concentrations for 24 h, followed by staining with 2 mM calcein-AM and propidium iodide. Fluorescence microscopy (Zeiss Axio Observer) was used to observe live cells (green fluorescence) and dead cells (red fluorescence).

*Hemolysis test*. For evaluation of hemolytic toxicity, mouse red blood cells were suspended in saline and adjusted to a hematocrit of 2%. Different concentrations of sExo samples (25, 50, 100, 200 and 300 μg/mL) were incubated with 0.5 mL of red blood cell suspension at 37 °C for 1 h. After centrifugation (3000 rpm, 5 min at room temperature), the supernatant was collected, and the absorbance at 540 nm was measured. The hemolytic percentage for each sample was calculated by using RO water as the 100% hemolysis control.

$$Hemolysis\;ratio\left(\%\right)=\frac{A_{s}-A_{n}}{A_{p}-A_{n}}$$

A_n_ and A_p_ represent the absorption at 540 nm of samples, the negative control (saline) and the positive control (RO), respectively.

*Coagulation test*. 50 μL of recalcified whole blood was added to different concentrations (25, 50, 100, 200 and 300 μg/mL) of sExos for solidification. RO water and saline were used as positive controls and negative controls, respectively. After 60 s, 10 mL of RO water was added. The samples were then collected and centrifuged (at 1500 rpm for 10 min) to obtain the upper serum layer, which contained hemoglobin. The absorbance of the supernatant was measured at 540 nm. The BCI was calculated as follows:

$$BCI\left(\%\right)=\frac{A_{s}}{A_{p}}\times 100\%$$

A_s_ is the absorbance of the sample, and A_p_ is the absorbance of the positive control.

### 2.9 In vivo safety study

To evaluate the *in vivo* safety, sExos (4 mg/kg) were administered intravenously to the mice. 24 h later, the mice were sacrificed, and their organs, including the heart, liver, spleen, lung, and kidney, were collected for Hematoxylin-Eosin (H&E) staining. In addition, mouse blood was centrifuged (2500 rpm, 5 min) to obtain serum for routine blood tests and analysis of liver and kidney function.

### 2.10 Establishment of the tMCAO model

Male C57BL/6J mice weighing 25-30 g were selected and anesthetized with isoflurane. The right external carotid artery (ECA) and internal carotid artery (ICA) were exposed. A dedicated thread embolus was inserted through the ECA until the blood flow in the middle cerebral artery (MCA) was occluded. After the ischemic condition was maintained for 1 h, the thread embolus was removed to restore blood flow. During surgery, cerebral blood flow changes were monitored in real time *via* the RFLSI III laser speckle imaging system (RFLSI III, RWD).

### 2.11 In vivo imaging

Cyanobacteria and sExo were injected into tMCAO mice, and the *in vivo* biodistribution was monitored in real-time *via* a spectral imaging system (Cailper PerkinElmer, USA) at 1, 3, 6, and 12 h. Subsequently, the mice were sacrificed, and the brains, hearts, livers, spleens, lungs, and kidneys were collected for imaging analysis. The average radiance efficiency of each tissue sample was quantitatively analyzed *via* Living Image software.

### 2.12 BBB penetration

BBB penetration by sExo was investigated by creating cranial windows for two-photon microscopy (A1RMP+, Nikon). Briefly, a 3 × 3 mm^2^ gap was opened above the infarct brain of each mouse, which was then covered with a coverslip and fixed with bone cement. When the mice recovered, tMCAO was performed, and the mice were treated with cyanobacteria and their sExos, followed by injection with 100 μL of FITC-dextran (2 mg/mL, 2000 KDa) to stain blood vessels. 30 min later, images were obtained *via* Z-stack scanning from 200 μm to 500 μm below the cortical surface.

### 2.13 In vivo therapy

Male C57BL/6J mice (25-30 g) subjected to tMCAO were randomly assigned to three groups (n = 5 per group): sham, MCAO, and sExo. At 0, 24, and 48 h post-ischemia, mice in the MCAO group received saline injections, whereas mice in the sExo group received sExo (4 mg/kg).

Therapeutic efficacy was evaluated at 72 h post-ischemia, including infarct volume assessment and histological analyses. In addition, long-term functional recovery was assessed by behavioral testing from day 0 to day 28 post-ischemia to determine sustained neurological improvement.

### 2.14 Triphenyltetrazolium chloride (TTC) staining

After the mice were euthanized, brain tissues were extracted and sectioned into five 2-mm-thick slices. The brain slices were immersed in 2% TTC solution and incubated at 37 °C for 30 min. Subsequently, the slices were fixed in 4% paraformaldehyde. Normal brain tissue appeared rose-red, whereas infarcted tissue was white. Quantitative analysis of the total and infarcted areas was performed *via* the ImageJ analysis system.

### 2.15 Histological staining

To assess the therapeutic effects of extracellular vesicles in stroke treatment, Nissl staining, Luxol Fast Blue (LFB) staining, and H&E staining were performed on brain tissues on day 3. Imaging analysis was performed *via* optical microscopy. In the LFB-stained sections, the blue-stained areas were identified as positive regions *via* Image-Pro Plus 6.0 software. The integrated optical density (IOD) and pixel area (AREA) of the positive regions in each image were analyzed, and the average optical density (AO) was calculated *via* the formula AO = IOD/AREA. The same analytical method was applied to the Nissl-stained images.

### 2.16 Immunofluorescence staining

On the third day after MCAO, the brain tissue was perfused and fixed with saline through the heart, followed by fixation with 4% paraformaldehyde. The brain tissue was sectioned into 20 μm coronal slices. The slices were incubated overnight at 4 °C with primary antibodies. The primary antibodies used included rabbit anti-NeuN (Abcam) and rabbit anti-NF-κB p65 (Bioss). After washing with PBS, the slices or cells were incubated at room temperature for 1 h with Alexa Fluor 488-conjugated goat anti-rabbit IgG. Nuclei were stained with DAPI (Sigma). TUNEL staining was performed according to the manufacturer's instructions (Solarbio, T2130). Finally, fluorescence signals were observed *via* a confocal laser scanning microscope (CLSM, Nikon Eclipse Ti).

### 2.17 ROS measurement

The brains were separated from the mice on day 3 after MCAO and cut into 20 μm frozen coronal sections. DHE staining was performed to detect ROS according to the manufacturer's protocol (Abcam). Images were obtained *via* confocal microscopy. The mean fluorescence density was quantified with ImageJ software.

### 2.18 Mitochondrial protection

During ischemia-reperfusion, excessive ROS can damage mitochondrial structures, resulting in a decrease in the mitochondrial membrane potential (Δψ). To observe mitochondrial morphology in the mouse brain, the mice were euthanized, and the brain tissues were harvested. The tissues were fixed and dehydrated, followed by staining with uranyl acetate. The processed samples were subsequently imaged *via* TEM. Mitochondrial morphological parameters, including perimeter, area, length, and width, were quantitatively analyzed *via* ImageJ software.

### 2.19 BBB permeability

Two-photon microscopy was used to assess the permeability of the BBB. After treatment, a cranial window was established on the infarcted side of the mouse head, and 100 μL of 4% Evans blue was injected *via* the tail vein. Leakage of Evans blue into damaged cerebral vessels was observed. To examine the microscopic structure of the BBB, the mice were euthanized, and the brain tissues were harvested. The tissues were fixed in 2% glutaraldehyde solution, dehydrated and embedded. Ultrathin sections were prepared and stained with uranyl acetate and lead citrate. Finally, the microstructure of the BBB was observed and imaged *via* transmission electron microscopy (JEM-1400FLASH, JEOL).

### 2.20 Brain water content

The mice were sacrificed to separate the ischemic side of the brain, which was weighed to obtain the wet weight and subsequently freeze-dried to determine the dry weight. The brain water content was calculated *via* the following formula:

$$Brain\;water\;content\;\left(\%\right)=\frac{wet\;weight-dry\;weight}{wet\;weight}\times 100\%$$

### 2.21 Western blot (WB) analysis

To detect protein expression in the infarcted brain tissue, mouse brain tissues were homogenized and lysed in RIPA buffer. The protein concentration was determined *via* a BCA protein assay kit. Equal amounts of total protein were separated *via* SDS-PAGE, transferred onto a PVDF membrane, and blocked with 5% nonfat dry milk. The membrane was incubated overnight at 4 °C with primary antibodies, including rabbit anti-NF-κB p65 (1:1000, Bioss), rabbit anti-IκBα (1:1000, Bioss), rabbit anti-VE-cadherin (1:1000, Abcam), rabbit anti-claudin-5 (1:1000, Abcam), rabbit anti-occludin (1:1000, Abcam), rabbit anti-ZO-1 (1:1000, Abcam), rabbit anti-Fasn (1:1000, Affinity), rabbit anti-Cptlα (1:1000, Proteintech), rabbit anti-ApoE (1:1000, Affinity), rabbit anti-Pparα (1:1000, Bioss), and rabbit anti-Lpl (1:1000, Affinity). The membrane was subsequently incubated with an HRP-conjugated secondary antibody (1:1000, Sigma) at room temperature for 1 h, after which the protein bands were detected *via* an enhanced chemiluminescence substrate. Finally, the band intensities were analyzed *via* ImageJ software.

### 2.22 Detection of inflammatory cytokines

The right brain tissue samples were harvested and weighed, followed by homogenization in lysis buffer at 4 °C. After homogenization, the samples were centrifuged at 1500×*g* for 20 min at 4 °C, and the supernatant was collected. Based on preliminary experimental results, the appropriate dilution factor of the supernatant was determined to ensure that the concentration did not exceed the maximum value of the standard curve. Finally, inflammatory cytokine detection was performed according to the instructions provided by the ELISA kit (Enze Biotechnology Co., Ltd., Shanghai).

### 2.23 qPCR analysis

To assess the expression levels of metabolism-related genes in the tissue, total RNA was extracted from the brains of treated mice and subjected to qPCR analysis. The extracted RNA was reverse-transcribed into cDNA *via* 5X All-In-One RT MasterMix. Relative expression levels of target genes were determined using the QuantStudio™ TM3 system with SsoAdvanced Universal SYBR Green Supermix, and the data were analyzed using the 2^−ΔΔCt^ method.

### 2.24 Behavioral testing

A series of behavioral experiments were conducted on days 0, 3, 7, 14, 21, and 28 after stroke to investigate the therapeutic effects of sExos on long-term neurological damage in stroke model mice. These tests include the mNSS, corner turning test, adhesive test, cylinder test, rotarod test, Barnes maze test and Morris water maze test.

### 2.25 Statistical analysis

Statistical analysis was performed *via* SPSS software. The differences between each group were analyzed using Student's *t-*test. Significant differences among multiple groups were analyzed by either one-way ANOVA or two-tailed Pearson correlation. All the data are presented as the means ± SDs. ∗*p* < 0.05, ∗∗*p* < 0.01, ∗∗∗*p* < 0.001, ns, not significant.

## 3 Results and discussion

### 3.1 Preparation and characterization of sExos

**Fig. 2:**
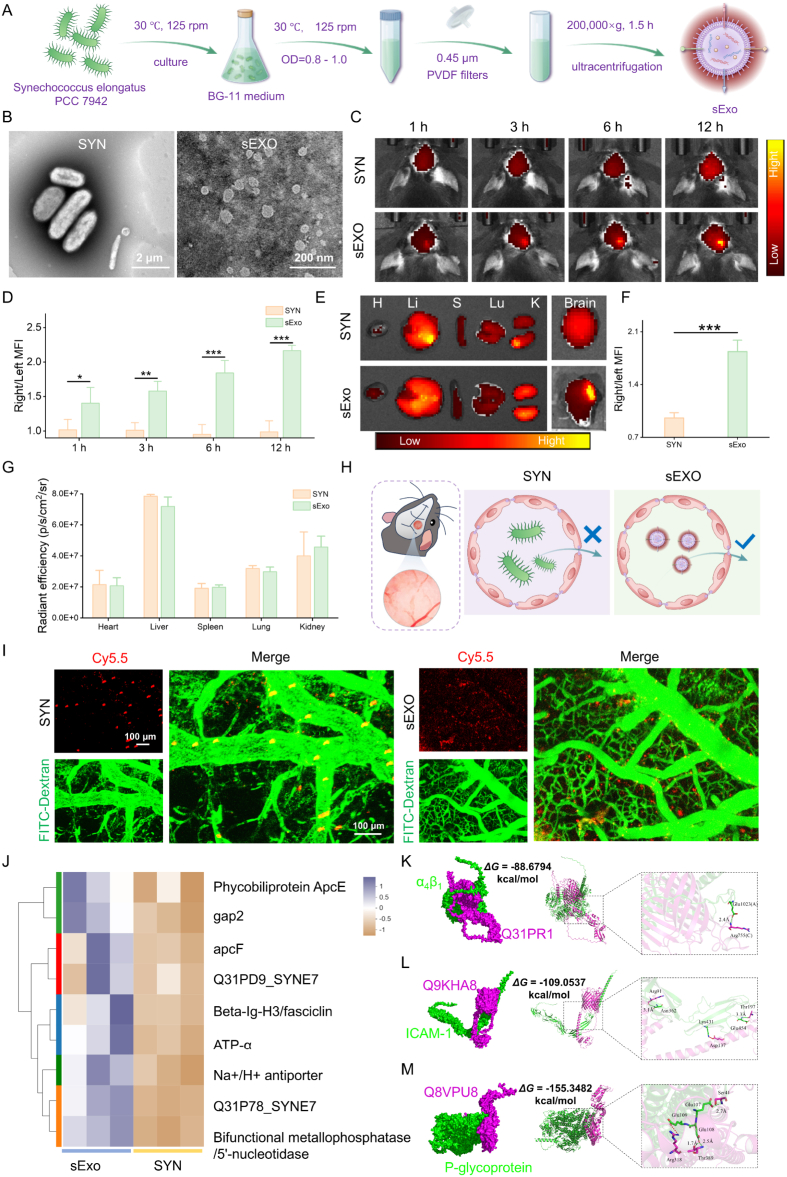
Fabrication and characterizations of sExo. (A) Schematic diagram of the preparation of sExo. (B) TEM image of SYN and sExo. (C) *In vivo* fluorescence imaging of SYN and sExos intravenously injected into tMCAO mice. (D) The average fluorescence intensity ratio between the right brain and the left brain in the mice. (E) *Ex vivo* fluorescence imaging of mouse organs in each group. H, heart; Li, liver; S, spleen; Lu, lung; K, kidney; B, brain. (F) Average radiation efficiency in each group on the basis of tissue fluorescence intensity (n = 3). (G) Comparison of the average fluorescence intensity between the SYN and sExo groups across major *ex vivo* mouse tissues. (H) The schematic shows the use of two-photon microscopy to assess the BBB penetration of SYN and sExo. A cranial window was created after fixing the mouse head on a brain locator, allowing direct observation of brain regions. (I) *In vivo* two-photon imaging showing the penetration of SYN and sExo from blood vessels into the brain parenchyma 30 min after intravenous injection in tMCAO mice. Red, Cy5.5 (sExo: Ex 644 nm/Em 655 nm); green, FITC-dextran (MW = 2000 KDa). (J) Expression of cyanobacteria-related proteins. (K) Three-dimensional binding model between integrin α_4_β_1_ (green) and Q31PR1 (magenta) (left), and schematic diagram of interface residue interactions (right). (L) Three-dimensional binding model between ICAM-1 (green) and Q9KHA8 (magenta) (left), and schematic diagram of interface residue interactions (right). (M) Three-dimensional binding model between P-gp (green) and Q8VPU8 (magenta) (left), and schematic diagram of interface residue interactions (right). One-way ANOVA, two-way ANOVA or two-tailed unpaired t-tests were used to calculate *p*-values. (∗*p* < 0.05, ∗∗*p* < 0.01, ∗∗∗*p* < 0.001, ns, not significant).

The sExos were isolated from the culture medium of cyanobacteria *via* ultracentrifugation [25,29] (Fig. 2A). A TEM image of the cyanobacteria, which have a fusiform shape, is shown in Fig. 2B. The extracted cyanobacterial sExos were analyzed using TEM, SEM, DLS and NTA. The results revealed that the isolated sExos had an average diameter of approximately 163 nm and displayed distinct vesicular structures (Fig. 2B and Fig. S1A–C). Notably, these sExos exhibited excellent colloidal stability during storage at 4 °C over 28 days, with minimal size variation, slightly reduced PDI, and a consistently maintained zeta potential around −14 mV, indicating preserved structural integrity and electrostatic stability (Fig. S1D).

*In vitro* cytotoxicity studies revealed that sExo, at a concentration of 300 μg/mL, cocultured with bEnd.3,PC-12, and SH-SY5Y cells exhibited excellent biocompatibility, with cell viability exceeding 85% for all three cell types (Fig. S2–S4). Meanwhile, sExo treatment significantly enhanced the antioxidant capacity of SH-SY5Y cells, as evidenced by an effective reduction in intracellular ROS levels (Fig. S5). Notably, ROS levels decreased in a dose-dependent manner with increasing concentrations of sExo, accompanied by a corresponding improvement in cell viability. These findings further indicate that sExo plays a protective role in alleviating oxidative stress-induced damage, thereby contributing to the maintenance of neuronal functional stability. Furthermore, the hemolysis rate of the sExo was less than 5% (Fig. S6), and the blood coagulation index (BCI) was comparable to that of the physiological saline group (Fig. S7), further demonstrating its excellent hemocompatibility.

Additionally, the potential toxicity of sExo to the heart, liver, spleen, lungs, and kidneys was investigated *via* H&E staining (Fig. S8). As shown in Fig. S8, no significant histopathological abnormalities were observed in either the sExo or saline groups. Serum levels of ALT, AST, ALP, CREA, UREA, and UA were measured to assess the impact of sExo on liver and kidney function. The results revealed no significant signs of liver or kidney toxicity in the sExo group compared with the saline group (Fig. S9). The results of routine blood examination of the different groups are shown in Fig. S10, and no significant changes in white blood cell (WBC) count, neutrophil (Gran) count, lymphocyte, or monocyte count were observed compared with those in the saline group, suggesting that sExo dose not induce inflammation *in vivo*. Furthermore, H&E staining of brain tissues from normal mice treated with sExo revealed no evidence of structural abnormalities, inflammatory infiltration, or necrosis (Fig. S11). In healthy mice, Laser speckle imaging confirmed that SYN injection did not affect blood flow or vascular patency (Fig. S12A and B), while sExo treatment did not alter platelet activation markers (CXCL4 and TXB2) (Fig. S12C), indicating favorable hemocompatibility. These results collectively demonstrate that sExo exhibits good biocompatibility and biosafety *in vivo*.

### 3.2 First discovery of sExos autofluorescence and BBB penetration for targeting ischemic brain

During *in vivo* imaging experiments using a transient middle cerebral artery occlusion (tMCAO) mouse model [30,31], we unexpectedly observed that intravenously administered SYN and sExo emitted stable and clearly visible autofluorescence signals within the NIR-I window (Fig. 2C), even in the absence of any exogenous fluorescent dye labeling. Notably, this autofluorescence was most prominent in the Cy5.5 channel. This finding suggests that sExo may intrinsically carry bio-components with near-infrared luminescent properties, thereby conferring strong potential for label-free *in vivo* imaging applications. Quantitative analysis further demonstrated that sExo exhibited pronounced brain accumulation in the MCAO model, with a time-dependent preference toward the ischemic hemisphere. At 12 h post-injection, the fluorescence intensity on the ipsilateral side reached 2.16 times that of the contralateral side (Fig. 2D), with a SNR as high as 17.2, indicating excellent imaging resolution and specificity. In contrast, the SYN group showed no significant fluorescence intensity difference between the two cerebral hemispheres. *Ex vivo* fluorescence imaging of major organs revealed that sExo predominantly accumulated in the brain (Fig. 2E and F), while moderate fluorescence signals were observed in the liver, lung, and kidney, and the heart and spleen exhibited comparatively lower signal intensities (Fig. 2E and G), suggesting a degree of inherent brain-targeting capability.

To further investigate the source and underlying mechanisms of the enhanced fluorescence signal of sExo in brain tissue, we performed a multi-level analysis combining intravital microscopic imaging and proteomic data. In a mouse model of MCAO, sExo was administered *via* tail vein injection, followed by labeling of cerebral vasculature with FITC-Dextran. Brain tissues were then imaged in situ 30 min post-injection using two-photon fluorescence microscopy (Fig. 2H and I). The results demonstrated that sExo was able to widely distribute across the brain parenchyma after crossing the BBB, with distinct intrinsic fluorescence signals observed in the Cy5.5 channel. In contrast, the SYN exhibited fluorescence signals confined to the vascular lumen, indicating a limited capacity to penetrate the BBB. This difference may be attributed to disparities in particle size and structural complexity between the two, as the nanoscale dimensions and membrane composition of sExo are more favorable for traversing the structural barriers of the BBB.

Based on the above observations that sExo can effectively penetrate brain tissue and generate distinct fluorescence signals in the NIR channel, we further investigated whether this fluorescence originates from intrinsic protein components. To this end, we conducted a quantitative proteomic analysis of SYN and sExo. A total of 1736 differentially expressed proteins were identified, among which 130 were significantly upregulated and 1606 downregulated in sExo compared to SYN (Fig. S13 and S14). Gene Ontology (GO) enrichment analysis revealed that the upregulated proteins were predominantly enriched in pathways related to membrane structure, membrane association, and transmembrane transport (Fig. S15), suggesting that sExo is enriched with specific membrane-associated functional proteins, which may contribute to its enhanced cellular internalization and tissue permeability.

Furthermore, among the proteins with comparable expression levels between sExo and SYN, several cyanobacteria-specific structural proteins were retained (Fig. 2J). Notably, the phycobiliprotein ApcE was consistently detected in both samples. Phycobiliproteins, which are unique photosynthetic pigment-protein complexes in cyanobacteria, contain covalently bound chromophores such as phycocyanobilin (PCB), with typical excitation wavelengths ranging from 495 to 650 nm, and some subtypes emitting fluorescence that extends into the NIR region [[32], [33], [34], [35]]. ApcE, serving as a core anchoring protein within the phycobilisome complex, plays a crucial role in the structural organization of light-harvesting complexes and in energy transfer. Importantly, ApcE itself exhibits characteristic fluorescence properties [36]. Previous studies have shown that chromophore-binding complexes involving ApcE can produce both excitation and emission signals in the range of approximately 516-660 nm [[36], [37], [38]], which closely aligns with the spontaneous fluorescence signals observed in the Cy5.5 channel (NIR region) in our study. Therefore, we speculate that ApcE is a key endogenous protein component responsible for the intrinsic NIR-I fluorescence exhibited by both sExo and its parental SYN cells.

Building on the confirmation that sExo can traverse the BBB and exhibit brain-specific accumulation with intrinsic fluorescence advantages, we further investigated the underlying mechanisms of BBB penetration at the molecular level using protein-protein molecular docking [39,40]. Specifically, based on the above proteomic sequencing data, we identified 15 commonly expressed membrane proteins with no significant differential expression in both SYN and sExo (Data. S1), as well as 15 sExo-specific membrane proteins (Data. S2). These were selected as candidate ligand proteins for molecular docking simulations with three representative categories of BBB-associated receptors: α_4_β_1_ integrin [41], intercellular adhesion molecule-1 (ICAM-1) [42], and P-glycoprotein (P-gp) [43,44]. Binding affinities for all protein–receptor pairs were quantitatively assessed by calculating their binding free energies. The top three protein–receptor complexes with the lowest binding free energies were selected for further structural analysis, including the generation of 2D interaction maps and 3D structural models. Among the commonly expressed membrane proteins, Q31N76 demonstrated a strong binding affinity with α_4_β_1_ integrin (ΔG = −74.24 kcal/mol) (Fig. S16A), forming multiple hydrophobic interactions, primarily with hydrophobic residues located on the inner region of the α_4_ subunit. This suggests that Q31N76 may mimic endogenous cell adhesion mechanisms to facilitate sExo translocation across the BBB. Although the interaction between Q31IA4 and ICAM-1 was relatively weaker (ΔG = −51.72 kcal/mol) (Fig. S16B), it still formed a stable hydrogen bonding network, indicating a potential cooperative role in cellular recognition or adhesion. Q31N76 also showed remarkably strong binding with P-gp (ΔG = −149.63 kcal/mol) (Fig. S16C), where its transmembrane domain stably embedded into the multispanning cavity of P-gp. This interaction may inhibit the efflux function of P-gp through competitive binding or steric blockade, thereby enhancing sExo uptake and transcytosis across the endothelial layer. Among the sExo-specific membrane proteins, Q31PR1 bound to α_4_β_1_ integrin with a binding free energy of −88.68 kcal/mol (Fig. 2K). The interface included multiple hydrogen bonds and hydrophobic contacts, indicating a highly stable interaction and suggesting potentially greater trans-BBB efficiency compared to shared membrane proteins. Q9KHA8 showed strong binding to ICAM-1 (ΔG = −109.05 kcal/mol) (Fig. 2L), forming a network of hydrogen bonds and electrostatic interactions. The binding pattern closely resembled that of leukocyte rolling adhesion, implying that sExo may acquire adhesive recognition capabilities that initiate endothelial uptake. The strongest binding was observed for Q8VPU8 with P-gp (ΔG = −155.35 kcal/mol) (Fig. 2M), which formed a stable three-dimensional interaction network with extensive contact across multiple functional domains of P-gp. This highlights Q8VPU8 as a key candidate for modulating BBB penetration of sExo. Collectively, these findings identify P-gp as the central target in the transcytosis of sExo across the BBB, with the sExo-specific protein Q8VPU8 potentially enhancing brain-targeted delivery through multiple mechanisms. Additionally, membrane proteins binding to α_4_β_1_ integrin and ICAM-1 may contribute synergistically to recognition, adhesion, and endothelial transport. Cell-based inhibitor assays further validated that sExo transcytosis across the BBB is energy-dependent and primarily mediated *via* receptor-driven endocytic pathways (Fig. S17). These results elucidate the potential molecular mechanisms underlying sExo traversal of the BBB, as well as provide theoretical and structural guidance for their application in targeted delivery for central nervous system diseases.

In summary, sExo demonstrates excellent BBB permeability and brain-targeting capability and possesses intrinsic NIR imaging potential owing to its endogenous fluorescence proteins, thereby enabling specific *in vivo* imaging without the need for exogenous labeling. This dual functionality highlights the unique advantage of sExo as both a natural optical probe and a brain-targeted delivery vector. Such features provide a distinctive molecular basis and promising application potential for the integration of diagnosis and therapy in CNS diseases.

### 3.3 Evaluation of the *in vivo* therapeutic effects of sExos

**Fig. 3:**
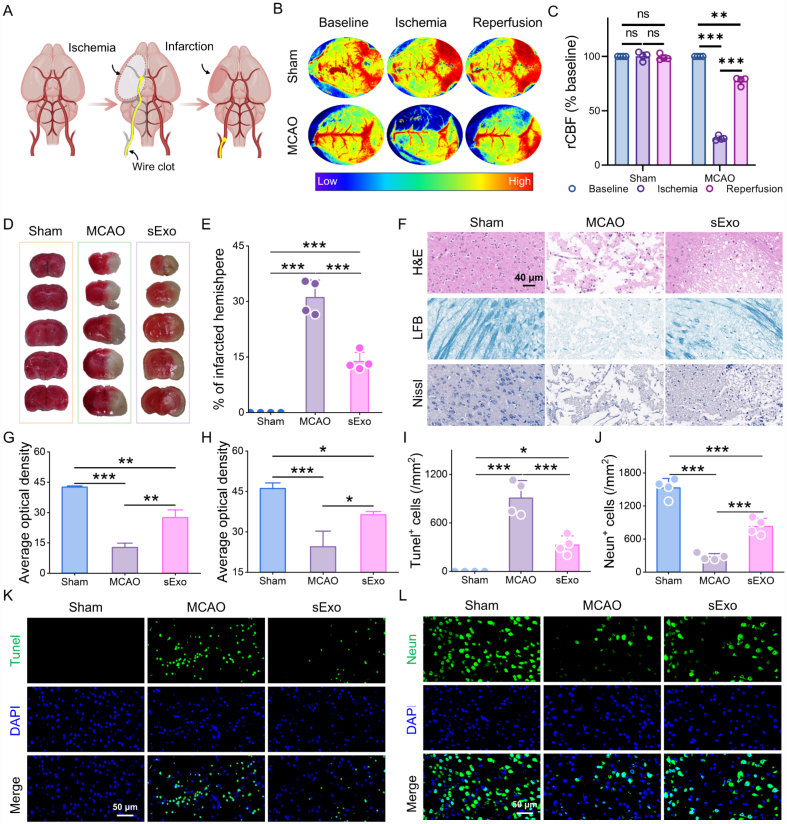
Assessment of the *in vivo* therapeutic efficacy of sExo. (A) Schematic diagram of the establishment of the tMCAO model. (B) Laser speckle contrast images from the sham and MCAO groups before surgery, during surgery, and after reperfusion. (C) Quantification of the rCBF in each group (n = 4). (D and E) TTC staining (D) and (E) infarct ratios of MCAO mice (n = 4). (F) Nissl, LFB, and H&E staining in different groups. (G and H) Average optical density in (G) LFB staining and (H) Nissl staining (n = 4). (K) Tunel staining of ischemic brain in different groups. Tunel-positive cells were stained green, and nuclei were stained with DAPI (blue). (I)Three days after MCAO, the Tunel-positive cells on the right side of the brain were counted. (L) Immunofluorescence staining of NeuN (green), a marker of neurons, in the damaged brains of mice 3 days after MCAO. (J) Three days after MCAO, the positive cell count in the right brain was determined (n = 4). ∗*p* < 0.05, ∗∗*p* < 0.01, ∗∗∗*p* < 0.001, ns, not significant.

After confirming the excellent BBB permeability and NIR imaging capability of sExo, we further investigated their potential therapeutic role in CNS disorders, with a particular focus on their effects on neural repair following brain tissue injury. To this end, a tMCAO model was established in mice using the intraluminal filament technique (Fig. 3A), mimicking the pathological process of ischemic stroke. Real-time monitoring of regional cerebral blood flow (rCBF) before and after ischemia was performed using laser speckle contrast imaging. As shown in Fig. 3B and C, prior to ischemia, cerebral blood flow in the ipsilateral hemisphere remained stable and comparable to baseline levels. Following occlusion, rCBF dropped to less than 30% of the baseline, indicating severe focal cerebral ischemia. During the reperfusion phase, blood flow recovered to approximately 80% of baseline, consistent with the criteria for successful ischemia-reperfusion modeling. These results not only confirm the successful establishment of the tMCAO model but also demonstrate high biological consistency among experimental subjects in terms of ischemic severity and reperfusion recovery, thereby providing a robust foundation for subsequent evaluation of therapeutic interventions.

Based on these findings, we systematically evaluated the neuroprotective and reparative effects of sExo in the tMCAO model. TTC staining revealed that MCAO surgery induced prominent cerebral infarction, with the infarct area accounting for 31.07 ± 4.71% of the total brain tissue. In contrast, treatment with sExo significantly reduced the infarct size to 13.80 ± 2.49% (Fig. 3D and E), indicating a marked tissue-protective effect. Histological analyses further corroborated these observations. H&E staining demonstrated extensive necrosis, structural disorganization, and vacuolation in the cortical and subcortical regions of the MCAO group, whereas the sExo-treated group largely preserved tissue integrity with no evident neuronal damage (Fig. 3F). Further analyses using LFB staining and Nissl staining demonstrated that the density of myelinated axonal fibers (Fig. 3F and G) and the number of Nissl bodies (Fig. 3F and H) in the ischemic regions were significantly higher in the sExo-treated group compared to the MCAO group. Furthermore, a significant reduction in mean optical density within the infarcted regions indicates that sExo treatment effectively mitigated demyelination and preserved neuronal structural integrity in response to ischemic insult.

In terms of apoptosis, TUNEL fluorescence staining showed substantial apoptotic signals in the cortical region of MCAO mice (Fig. 3I and K), whereas the sExo group exhibited a notable reduction in apoptotic cells, indicating its anti-apoptotic effects at the cellular level. Furthermore, immunostaining for neuron-specific markers revealed that sExo treatment significantly alleviated neuronal injury and preserved structural integrity of brain tissue (Fig. 3J and L), collectively supporting its multifaceted role in post-stroke neural repair. In summary, sExo demonstrated robust neuroprotective and tissue-reparative effects in the tMCAO model. Its intervention significantly reduced cerebral infarct volume, mitigated neuronal structural damage, decreased cellular apoptosis, and preserved myelin integrity to a considerable extent. These findings provide preliminary evidence of the biological activity of sExo in ischemic brain injury and lay an experimental foundation for further mechanistic studies and its potential therapeutic application in CNS diseases.

### 3.4 Protective effects of sExo on BBB structure and function after ischemic stroke

**Fig. 4:**
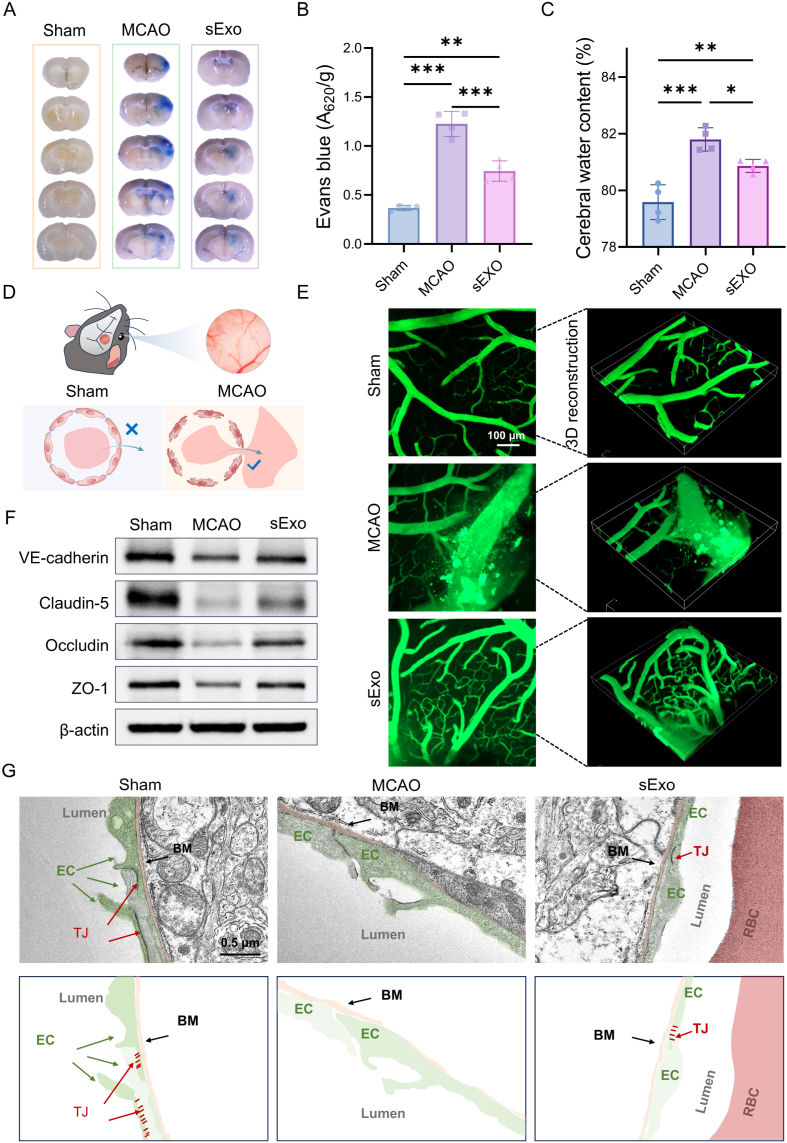
sExo-mediated protection of BBB structure and function following ischemic stroke. (A) Digital images showing the extravasation of Evans blue dye from the damaged BBB. (B) Quantitative analysis of the Evans blue content in the different groups (n = 4). (C) Brain water content in different groups. (n = 4). (D) The process of detecting the integrity of the BBB by two-photon microscopy is schematically demonstrated. (E) *In vivo* two-photon imaging showing FITC-dextran (MW = 3500 Da) penetrating damaged vessels into the brain parenchyma. (F) WB analysis of VE-cadherin, Claudin-5, occludin and ZO-1 in the ischemic brains of different mice. (G) TEM images of BBB in different groups. TJ, tight junction; BM, basement membrane. One-way ANOVA was used to calculate *p* values (∗*p* < 0.05, ∗∗*p* < 0.01, ∗∗∗*p* < 0.001).

Building upon the previously observed neuroprotective effects, we further evaluated the potential role of sExo in promoting vascular remodeling following ischemic stroke. Evans Blue dye was employed to assess changes in BBB permeability, which can penetrate a compromised BBB but does not cross an intact barrier. No Evans Blue extravasation was detected in the Sham group, whereas significant dye leakage was observed in the brains of MCAO animals (Fig. 4A), indicating severe BBB disruption induced by ischemic injury, allowing dye infiltration into the brain parenchyma. Notably, sExo treatment resulted in a significant reduction of Evans Blue extravasation area by 39.18% on average (Fig. 4B), suggesting effective attenuation of BBB damage by sExo.

To further evaluate cerebral edema, brain water content was measured across groups. The results demonstrated a marked increase in brain water content to 81.79 ± 0.41% following MCAO surgery, indicative of pronounced edema; this value was significantly reduced to 80.86 ± 0.23% after sExo administration (Fig. 4C), reflecting alleviation of edema. Subsequently, two-photon microscopy was utilized for real-time observation of vascular permeability within the infarcted region. Specifically, a 3 × 3 mm cranial window was established on the mouse skull, and FITC-dextran was administered intravenously *via* the tail vein to monitor vascular leakage (Fig. 4D). Pronounced perivascular fluorescence leakage was evident in the infarct area of MCAO mice, which was substantially suppressed following sExo treatment (Fig. 4E).

At the molecular level, WB analysis revealed that MCAO induced downregulation of key tight junction proteins, including VE-cadherin, Claudin-5, Occludin, and ZO-1 [45]. Importantly, sExo treatment significantly reversed the loss of these proteins (Fig. 4F and Fig. S18), further supporting its protective effect on BBB structural integrity. To validate these findings at the ultrastructural level, TEM was performed to examine cerebral microvascular morphology in ischemic hemispheres. TEM images from the MCAO group exhibited characteristic BBB damage features, such as basement membrane dissolution and tight junction disruption (Fig. 4G). In contrast, the sExo-treated group preserved relatively intact endothelial junctions. Collectively, these results demonstrate that sExo treatment partially alleviates structural and functional BBB impairments following cerebral ischemia, as evidenced by reduced vascular leakage, mitigated edema, and partial restoration of tight junction proteins. These changes suggest a protective role of sExo on the cerebral microvasculature after ischemic injury.

### 3.5 Functional recovery of neurological behavior induced by sExo

**Fig. 5:**
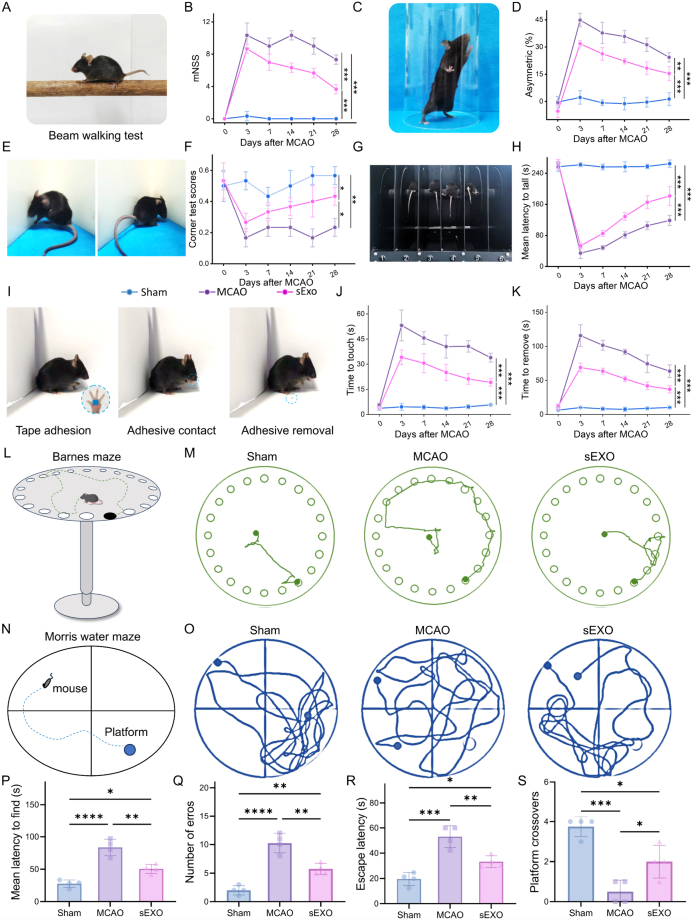
Long-term neurological functional recovery in tMCAO mice following sExo treatment. (A) Representative photo of the beam balance test. (B) mNSS, which includes motor, sensory acuity, balance, and reflex function assessments. (n = 4). (C) Representative photo of the cylinder test. (D) Forelimb asymmetry rate measured in the cylinder test. (n = 4). (E) Representative image of the corner turn test. (F) Laterality indices measured in the corner turn test. (n = 4). (G) Representative photo of the rotarod test. (H) Mean latency to fall during the rotarod test. (n = 4). (I) Schematic illustration of the adhesive test. (J) Time to contact the tape in the adhesive test. (n = 4). (K) Time to remove the tape in the adhesive test. (n = 4). (L) Schematic of Barnes maze test. (M) Representative images of walking path in different groups at day 28 after MCAO. (N) Schematic of the Morris water maze test. (O) Representative images of the swimming paths of the different groups on day 28 after MCAO. (P) Number of errors and (Q) latency to find the escape hole in the Barnes maze test. (n = 4). (R) Escape latency and (S) platform crossovers in the Morris water maze test. (n = 4). One-way or two-way ANOVA was used to calculate *p* values (∗*p* < 0.05, ∗∗*p* < 0.01, ∗∗∗*p* < 0.001, ns, not significant).

To further investigate the effects of sExo treatment on long-term neurological recovery in post-stroke mice, we conducted a series of behavioral assessments focusing on sensorimotor function and spatial cognitive ability. The results demonstrated that sExo significantly improved neurological performance in MCAO mice across multiple tests. In the modified neurological severity score (mNSS) assessment [46], sExo treatment markedly reduced neurological deficits, with the score decreasing from 8.67 in the MCAO group to 3.67 on day 28 (Fig. 5A and B), indicating an overall improvement in neurological function.

Results from the cylinder test showed that the forelimb use symmetry in the sExo-treated mice was significantly improved, approaching the level observed in the sham group (Fig. 5C and D). In the corner test, the lateralization index in the sExo group increased from 0.26 to 0.4, indicating a notable improvement compared to the MCAO group (Fig. 5E and F). In the rotarod test, sExo treatment significantly prolonged the time mice remained on the rotating rod, with the average latency to fall on day 28 reaching 1.53 times that of the MCAO group (Fig. 5G and H), suggesting enhanced recovery of motor coordination.

In the adhesive removal test, sExo treatment significantly reduced the time required for mice to detect and remove the adhesive from their forepaws, indicating improvements in somatosensory function and fine motor control (Fig. 5I–K). To evaluate long-term spatial cognitive function, the Barnes maze test was conducted (Fig. 5L). Results showed that mice in the sExo-treated group made fewer errors when locating the target hole (Fig. 5M), exhibited shorter latency to locate the target (Fig. 5P), and had fewer incorrect attempts (Fig. 5Q), suggesting enhanced cognitive learning abilities.

Subsequently, the Morris water maze test was conducted to further validate the above findings (Fig. 5N). Mice in the sExo-treated group exhibited more efficient and concise escape trajectories during the learning phase (Fig. 5O), demonstrated shorter average escape latency to locate the platform (Fig. 5R), and showed a significant increase in the number of platform crossings (Fig. 5S), further supporting the beneficial effects of sExo on spatial learning and memory. Additionally, body weight monitoring revealed that MCAO mice experienced a marked weight loss in the early post-stroke stage, whereas sExo treatment significantly accelerated body weight recovery (Fig. S19), indirectly indicating an improvement in overall physiological status. Survival analysis showed that sExo treatment markedly increased the survival rate of mice, reaching 1.67 times that of the MCAO group (Fig. S20). Collectively, these results demonstrate that sExo treatment exerts multifaceted neuroprotective effects by enhancing motor coordination, improving somatosensory function, and augmenting spatial cognitive performance, thereby significantly promoting long-term neurological recovery in MCAO mice.

### 3.6 Neuroprotective mechanisms of sExo

Following stroke, the ischemia-reperfusion process induces excessive generation of ROS [47]. ROS promote the degradation of cytoplasmic IκBα *via* oxidation, thereby relieving its inhibitory effect on the NF-κB p65 subunit and facilitating the translocation of NF-κB p65 into the nucleus. Within the nucleus, NF-κB p65 binds to specific DNA sequences to activate the transcription of pro-inflammatory cytokines such as IL-6 and TNF-α, further amplifying the inflammatory response. ROS, together with IκBα degradation, NF-κB nuclear translocation, and pro-inflammatory gene expression, form a positive feedback loop that perpetuates brain tissue damage [48,49]. Therefore, ROS play a central regulatory role in the activation of the NF-κB pathway and serve as key mediators of post-stroke inflammation.

**Fig. 6:**
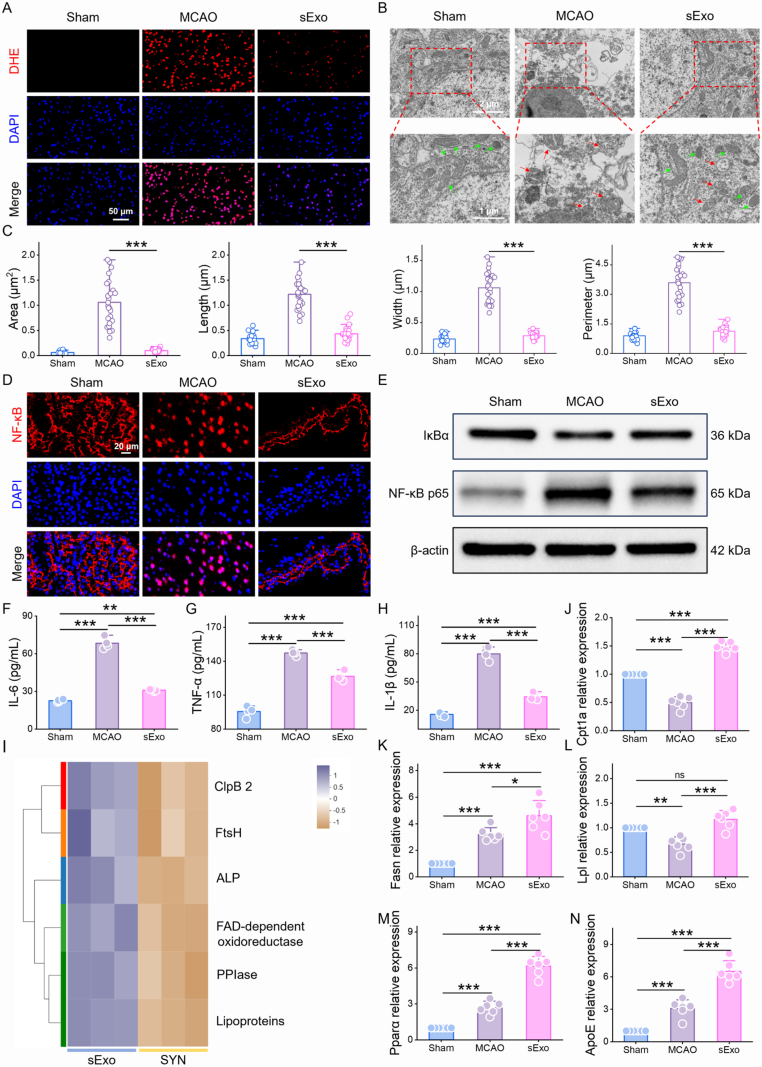
Neuroprotective mechanisms of sExo: inhibition of the ROS-NF-κB pathway and regulation of lipid metabolism. (A) Representative images of DHE-stained samples from different groups. (B) Representative TEM images showing the mitochondrial morphology of cells in the ischemic brain. Green arrows, normal mitochondria; red arrows, damaged mitochondria. (C) Quantification of mitochondrial morphological parameters of ipsilateral brain neurons in mice in different treatment groups. (n = 26). (D) Representative immunofluorescence images showing NF-κB (red) in the mouse brain. Nuclei were stained with DAPI (blue). (E) WB analysis of IκBα and NF-κB p65 in the ischemic brains of different mice. (F to H) IL-6, TNF-α and IL-1β levels in mouse brains were measured *via* ELISA. (n = 4). (I) Anti-inflammatory and antioxidant-related protein expression. (J to N) qRT-PCR analysis of Cpt1, Fans, Lpl, Pparα and ApoE in different groups. One-way ANOVA was used to calculate *p* values (∗*p* < 0.05, ∗∗*p* < 0.01, ∗∗∗*p* < 0.001).

To validate this pathway, ROS expression was assessed using immunofluorescence staining with the DHE probe. The results demonstrated that sExo treatment significantly reduced ROS levels in the brain (Fig. 6A and Fig. S21A). Consistently, quantitative analysis by ELISA further confirmed a marked elevation of ROS levels in the MCAO group compared with the sham group, which was significantly attenuated following sExo treatment (Fig. S21B). Excessive ROS can damage mitochondrial structure and induce functional impairment, ultimately leading to apoptosis. TEM revealed mitochondrial swelling, vacuolization, and cristae disruption in the brain tissue of the MCAO group, accompanied by significant increases in mitochondrial dimensions, including length, width, perimeter, and area (Fig. 6B and C). Following sExo treatment, mitochondrial morphology was markedly restored, indicating its efficacy in attenuating ischemia-reperfusion-induced mitochondrial damage.

Further analysis of IκBα degradation and NF-κB p65 nuclear translocation was conducted using immunofluorescence staining and WB. Immunofluorescence revealed a significant increase in nuclear NF-κB p65 signal in the brain tissue of MCAO mice (Fig. 6D), indicating that cellular stress promotes NF-κB activation. Treatment with sExo markedly reduced the nuclear NF-κB p65 fluorescence, with the signal predominantly localized in the cytoplasm, suggesting inhibition of nuclear translocation. WB analysis confirmed that sExo significantly increased cytoplasmic IκBα expression (Fig. 6E and Fig. S22), further blocking NF-κB pathway activation and suppressing the upregulation of pro-inflammatory cytokines including IL-6, TNF-α, and IL-1β (Fig. 6F–H).

Based on previous proteomic analyses of SYN and sExo, sExo is enriched with multiple specific proteins exhibiting anti-inflammatory, antioxidant, and anti-apoptotic functions (Fig. 6I). Notably, Peptidylprolyl Isomerase regulates NF-κB activity [50], thereby reducing the release of pro-inflammatory cytokines; Alkaline Phosphatase [51] and Lipoproteins [52] similarly suppress inflammatory responses; Chaperone Protein ClpB 2 is involved in the regulation of apoptosis [53]; while FAD-dependent Oxidoreductase and ATP-dependent Zinc Metalloprotease FtsH contribute to the reduction of ROS generation [54], protecting neurons from oxidative stress-induced damage. The synergistic action of these proteins likely underpins the neuroprotective effects exerted by sExo.

Additionally, we assessed the expression changes of genes related to lipid metabolism. sExo treatment significantly upregulated the mRNA levels of Cpt1a, Fasn, ApoE, Lpl, and Pparα (Fig. 6J–N), thereby promoting fatty acid oxidation and synthesis and improving lipid metabolic homeostasis in brain tissue. Furthermore, WB analysis confirmed that the protein expression trends were consistent with the qPCR results (Fig. S23A and B), and metabolite analysis revealed that TG levels were elevated while FFA levels were reduced in the MCAO group, whereas sExo treatment markedly decreased TG and restored FFA levels (Fig. S23C), indicating alleviation of lipid accumulation and recovery of fatty acid metabolism. Specifically, Cpt1a facilitates the transport of fatty acids into mitochondria for oxidation [55], Fasn maintains fatty acid synthesis [56], ApoE participates in lipid transport [57], Lpl promotes fatty acid release [58], and Pparα regulates fatty acid metabolism and oxidation [59]. This mechanism contributes to the restoration of metabolic homeostasis in neural cells and alleviates post-stroke brain tissue damage. In summary, sExo treatment mitigates ischemic brain injury and promotes neurological recovery by suppressing the ROS-NF-κB signaling pathway to attenuate inflammation and by modulating lipid metabolism to facilitate brain tissue repair.

## 4 Conclusion

In summary, this study is the first time to reveal the unique advantages of sExos in the diagnosis and treatment of stroke, particularly their ability to cross the BBB, achieve high SNR imaging, and exert neuroprotective effects. Unlike conventional brain-targeting probes that require exogenous labeling, sExos leverage the endogenous fluorescent protein phycobiliprotein ApcE to enable high-resolution, label-free imaging within the NIR-I window, significantly enhancing the visualization of ischemic lesions. In addition, sExos exhibit efficient BBB penetration and lesion-specific accumulation. During the acute phase of stroke, they modulate lipid metabolism, inhibit NF-κB signaling, alleviate oxidative stress and neuroinflammation, and induce protective cellular responses that contribute to neurological recovery. Toxicological and immunological assessments further confirm their excellent biocompatibility and biosafety *in vivo*. Collectively, sExos integrate intrinsic near-infrared autofluorescence with therapeutic potential, offering a multifunctional platform that bridges diagnosis and intervention. This work presents a promising, precise, and noninvasive strategy for the integrated management of stroke and other central nervous system disorders, with strong potential for clinical translation.

## Ethics approval and consent to participate

All animal experiments were approved by the Ethics Committee for Animal Research, Institutional Animal Care and Use Committee of Southwest Jiaotong University (Approval number: SWJTU-2403-NSFC(079)).

## CRediT authorship contribution statement

**Jingmei Pan:** Writing – review & editing, Writing – original draft, Methodology, Investigation, Formal analysis, Data curation, Conceptualization. **Yayun Wang:** Writing – review & editing, Writing – original draft, Methodology, Investigation, Formal analysis, Data curation, Conceptualization. **Yikun Feng:** Writing – review & editing, Writing – original draft, Project administration, Methodology. **Jiaoyang Wang:** Writing – review & editing, Writing – original draft, Project administration, Methodology. **Qiongya Huang:** Project administration, Methodology. **Lei Yan:** Project administration, Methodology. **Xiaobo Zhou:** Project administration, Methodology. **Huili Sun:** Supervision, Conceptualization. **Huaiyu Wang:** Supervision, Conceptualization. **Qu Wei:** Supervision, Conceptualization.

## Data availability statement

The authors declare that all other data supporting the findings of this study are available within the paper and Supplementary Materials.

## Declaration of competing interest

All authors declare that there are no competing interests.
