# Supplementary material for: Cyanobacteria-derived near-infrared autofluorescent exosomes enabling synergistic brain lesion imaging and neuroprotection
Source: Bioact Mater. 2026 Apr 20;63:718–31. doi: 10.1016/j.bioactmat.2026.04.027 (PMC13123330; doi:10.1016/j.bioactmat.2026.04.027)
Supplement: Multimedia component 1 [file mmc1.docx]

Supporting Information

**Cyanobacteria-derived near-infrared autofluorescent exosomes enabling synergistic brain lesion imaging and neuroprotection**

**Supplementary Figure Captions:**

**Supplementary** Fig. S1 **|** (A) SEM images, size distributions (B) and NTA (C) of sExos. (D) Colloidal stability of nanoparticles over 28 days as measured by DLS.

**Supplementary Fig. S2 |** (A) Live fluorescence staining and (B) cell viability data showing the *in vitro* cytotoxicity of bEnd.3 cells treated with different concentrations of sExos (n = 5).

**Supplementary Fig. S3 |** (A) Live fluorescence staining and (B) cell viability data showing the *in vitro* cytotoxicity of PC-12 cells treated with different concentrations of sExos (n = 5).

**Supplementary Fig. S4 |** (A) Live fluorescence staining and (B) cell viability data showing the *in vitro* cytotoxicity of SH-SY5Y cells treated with different concentrations of sExos (n = 5).

**Supplementary Fig. S5 |** (A) Representative fluorescence images of intracellular ROS in SH-SY5Y cells, indicating the oxidative stress levels under different treatment conditions. (B) Semi-quantitative analysis of ROS fluorescence intensity, based on statistical evaluation of intracellular ROS levels derived from fluorescence imaging. (C) Cell viability of SH-SY5Y cells under different treatment conditions, assessing the effect of sExo on cellular survival.

**Supplementary Fig. S6 |** (A) Hemolytic ratio of sExo at different concentrations. (B) Photographs of sExo hemolysis at different concentrations.

**Supplementary Fig. S7 |** Evaluation of total blood coagulation *in vitro* with different concentrations of sExos (n = 3).

**Supplementary Fig. S8 |** H&E staining of the heart, liver, spleen, lungs and kidneys of the mice.

**Supplementary Fig. S9 |** Blood biochemistry indices in different groups (n = 3).

**Supplementary Fig. S10 |** The contents of white blood cells (WBCs). (A), lymphocytes (B), monocytes (C), and neutrophils (D) in mouse blood (n = 3).

**Supplementary Fig. S11.** H&E staining of the brain of the mice.

**Supplementary** Fig. S12. (A) Laser speckle imaging showing blood flow dynamics in the vasculature. (B) Quantitative rCBF analysis. (C) ELISA detection of CXCL4 and TXB2 levels.

**Supplementary Fig. S13 |** Differences in protein expression between SYN and sExo.

**Supplementary Fig. S14 |** The heatmap shows sExo compared with SYN-upregulated proteins.

**Supplementary Fig. S15 |** GO enrichment analysis of upregulated proteins in the sExo group.

**Supplementary Fig. S16 |** (A) Three-dimensional binding model between integrin α_4_β_1_ (green) and Q31N76 (magenta) (left), and schematic diagram of interface residue interactions (right). (B) Three-dimensional binding model between ICAM-1 (green) and Q31IA4 (magenta) (left), and schematic diagram of interface residue interactions (right). (C) Three-dimensional binding model between P-gp (green) and Q31N76 (magenta) (left), and schematic diagram of interface residue interactions (right).

**Supplementary** Fig. S17. Inhibitor-based analysis of endocytic pathways involved in sExo transcytosis across the BBB.

**Supplementary Fig. S18 |** Protein expression levels of (A) Claudin-5, (B) Occludin, (C) ZO-1 and (D) VE-cadherin in each group (n = 3). One-way ANOVA was used to calculate *P* values **P* < 0.05, ***P* < 0.01, ****P* < 0.001, ns, not significant).

**Supplementary Fig. S19 |** Changes in the weights of the mice in the different treatment groups (n =4). Two-way ANOVA was used to calculate *P* values (**P* < 0.05, ***P* < 0.01, ****P* < 0.001, ns, not significant).

**Supplementary Fig. S20 |** Survival rates of the differently treated mice 28 days after tMCAO (n = 10).

**Supplementary** Fig. S21. (A) Quantitative analysis of DHE staining. n=4. (B) ROS levels in mouse brains were measured *via* ELISA. (n=4).One-way ANOVA was used to calculate *P* values (**P* < 0.05, ***P* < 0.01, ****P*< 0.001, ns, not significant).

**Supplementary Fig. S22 |** Protein expression levels of (A) IκBα and (B) NF-κB p65 in each group (n = 3). One-way ANOVA was used to calculate *P* values (**P* < 0.05, ***P* < 0.01, ****P* < 0.001, ns, not significant).

**Supplementary** Fig. S23. (A) WB analysis of Fasn, Cpt1α, Lpl, Pparα and ApoE in the ischemic brains of different mice. (B) Protein expression levels were measured in each group (n = 3). (C) ELISA detection of TG and FFA levels. One-way ANOVA was used to calculate *P* values (**P* < 0.05, ***P* < 0.01, ****P* < 0.001, ns, not significant).





**Supplementary** Fig. S1. (**A**) SEM images, size distributions **(B)** and NTA **(C)** of sExos. **(D)** Colloidal stability of nanoparticles over 28 days as measured by DLS.


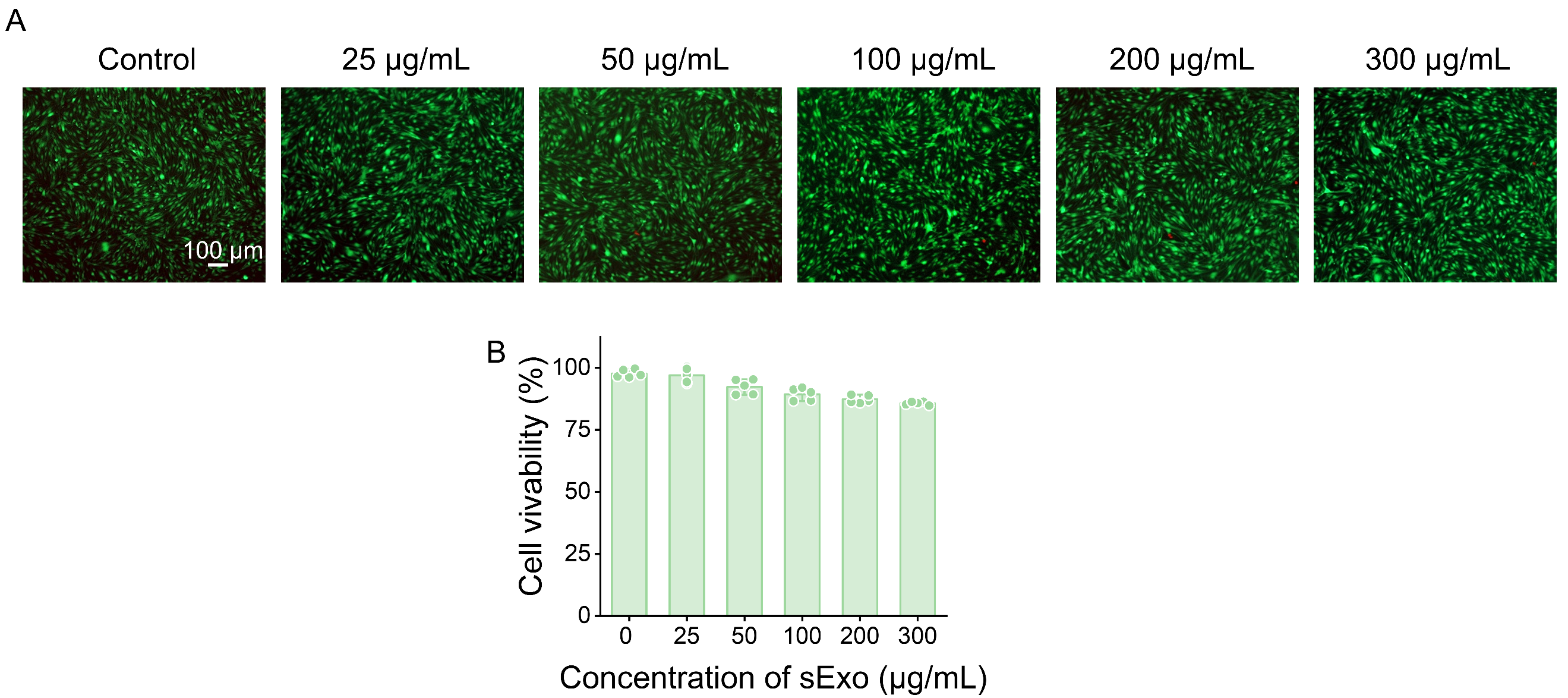


**Supplementary** Fig. S2. (**A**) Live fluorescence staining and **(B)** cell viability data showing the *in vitro* cytotoxicity of bEnd.3 cells treated with different concentrations of sExos (n = 5).


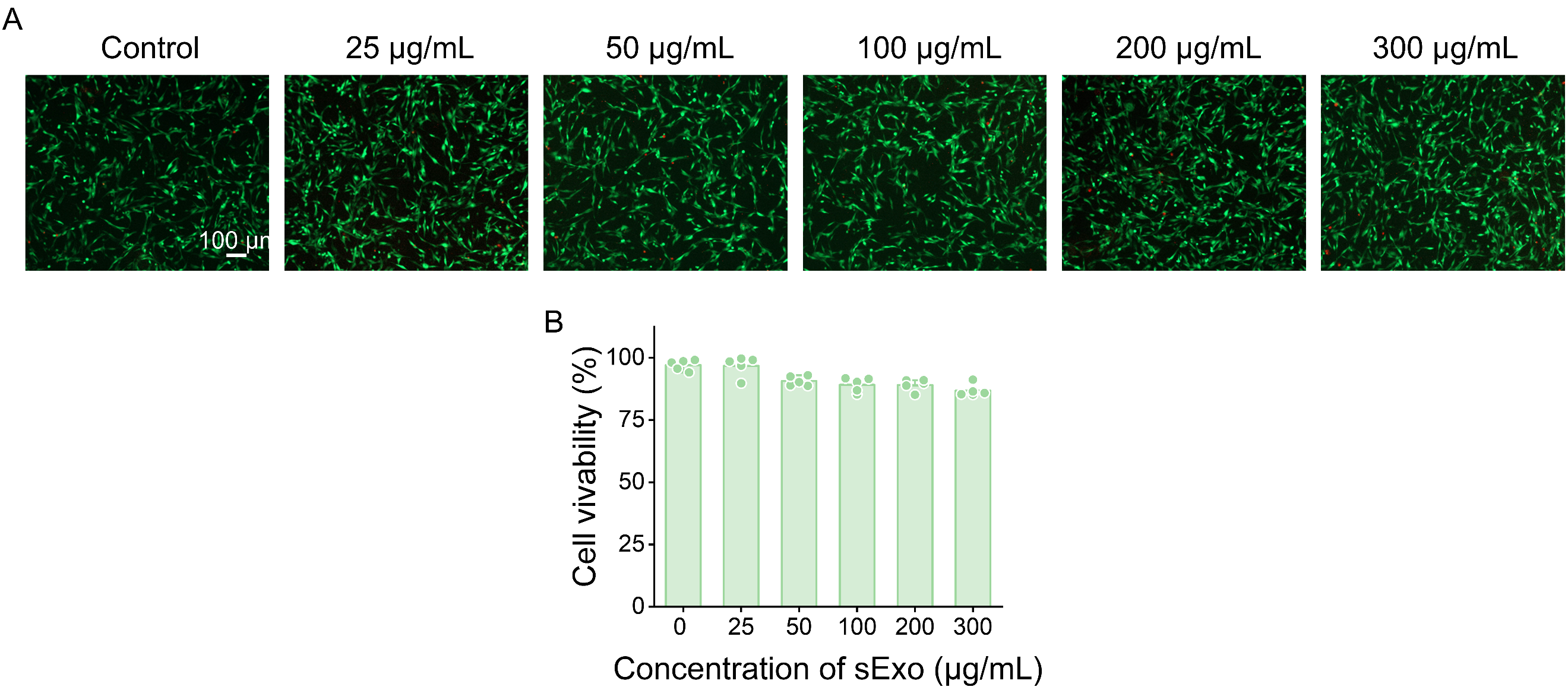


**Supplementary** Fig. S3. (**A**) Live fluorescence staining and **(B)** cell viability data showing the *in vitro* cytotoxicity of PC-12 cells treated with different concentrations of sExos (n = 5).





**Supplementary** Fig. S4. (**A**) Live fluorescence staining and **(B)** cell viability data showing the *in vitro* cytotoxicity of SH-SY5Y cells treated with different concentrations of sExos (n = 5).





**Supplementary** Fig. S5. (**A**) Representative fluorescence images of intracellular ROS in SH-SY5Y cells, indicating the oxidative stress levels under different treatment conditions. **(B)** Semi-quantitative analysis of ROS fluorescence intensity, based on statistical evaluation of intracellular ROS levels derived from fluorescence imaging. **(C)** Cell viability of SH-SY5Y cells under different treatment conditions, assessing the effect of sExo on cellular survival.


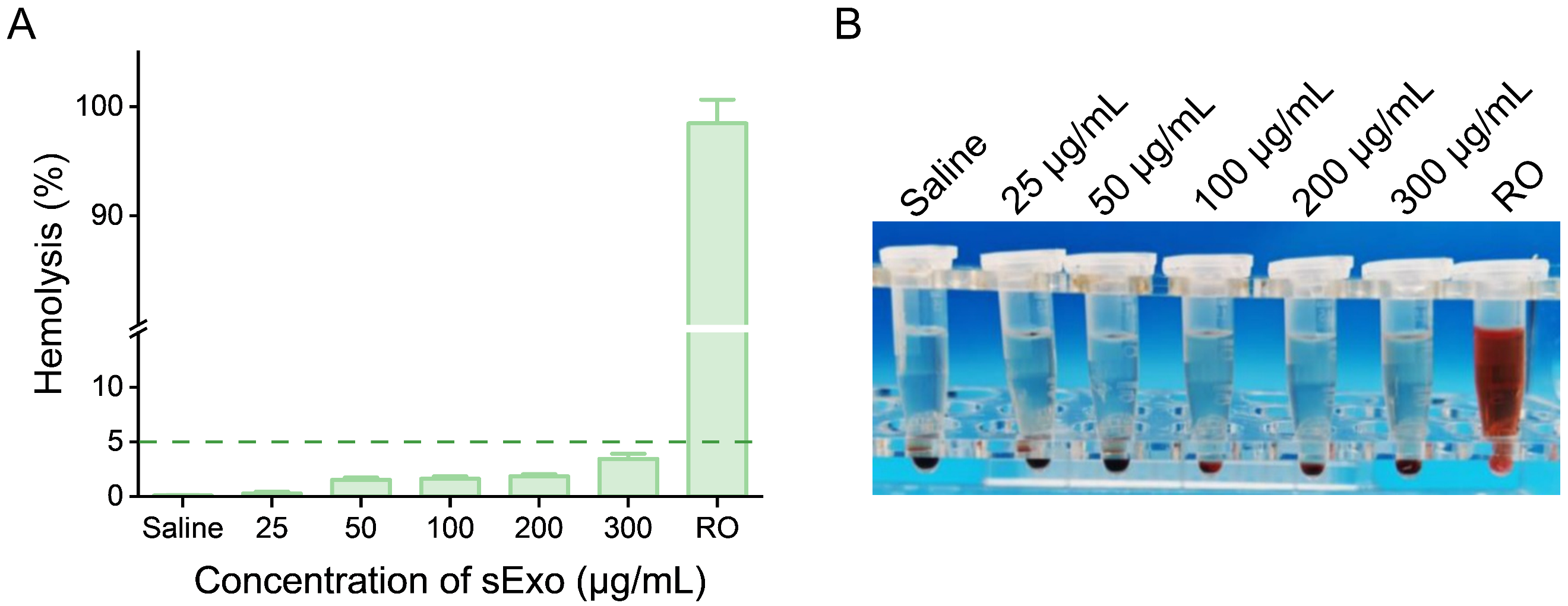


**Supplementary** Fig. S6. (**A**) Hemolytic ratio of sExo at different concentrations. **(B)** Photographs of sExo hemolysis at different concentrations.


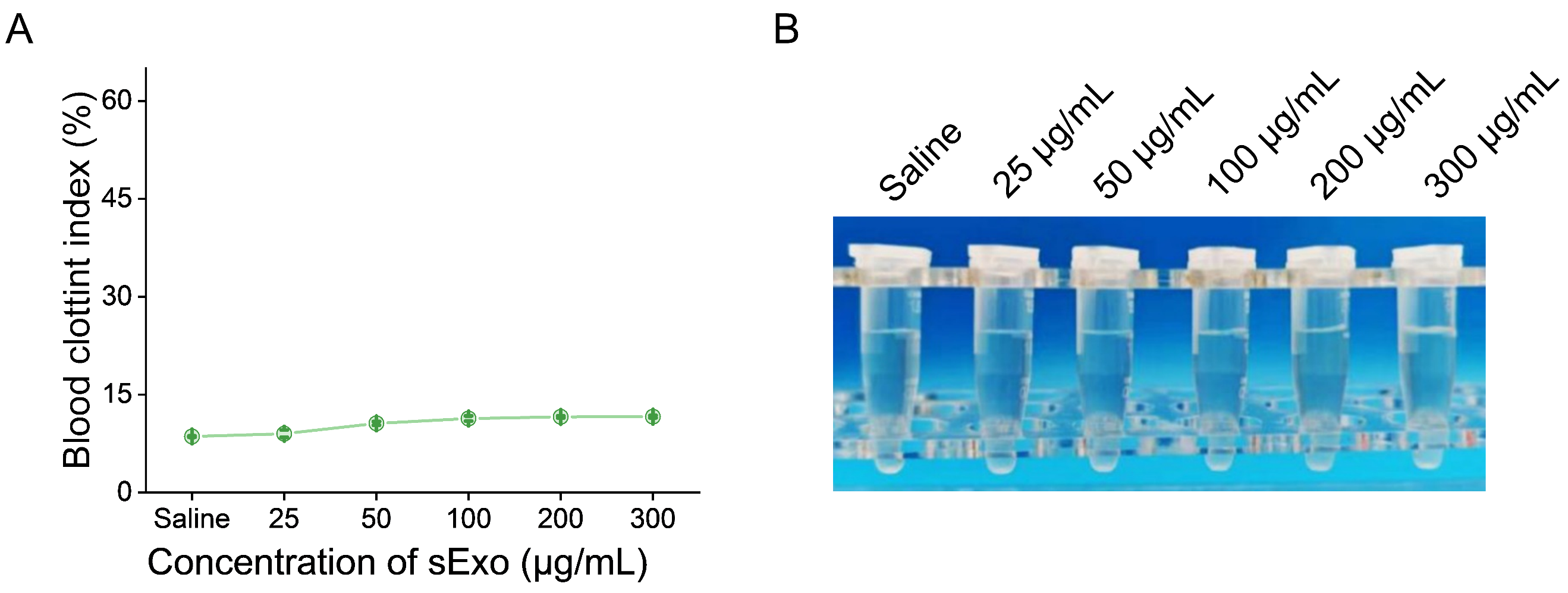


**Supplementary** Fig. S7. Evaluation of total blood coagulation *in vitro* with different concentrations of sExos (n = 3).


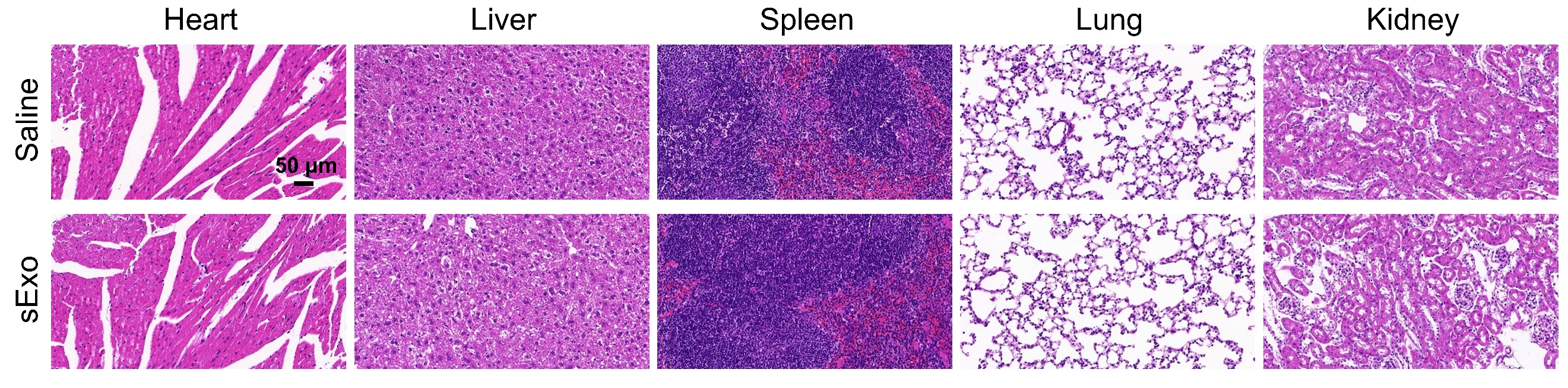


**Supplementary** Fig. S8. H&E staining of the heart, liver, spleen, lungs and kidneys of the mice.


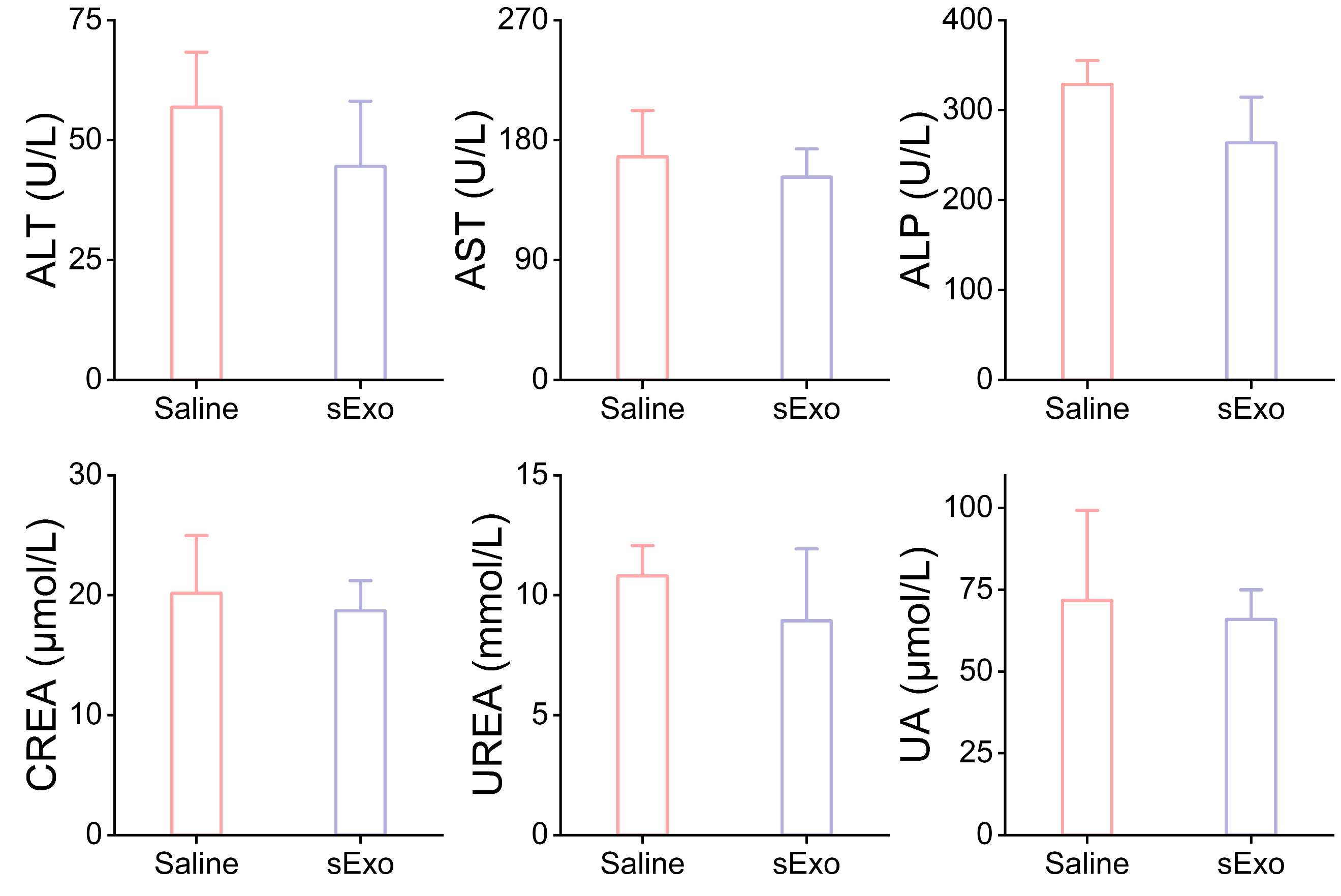


**Supplementary** Fig. S9. Blood biochemistry indices in different groups (n = 3).


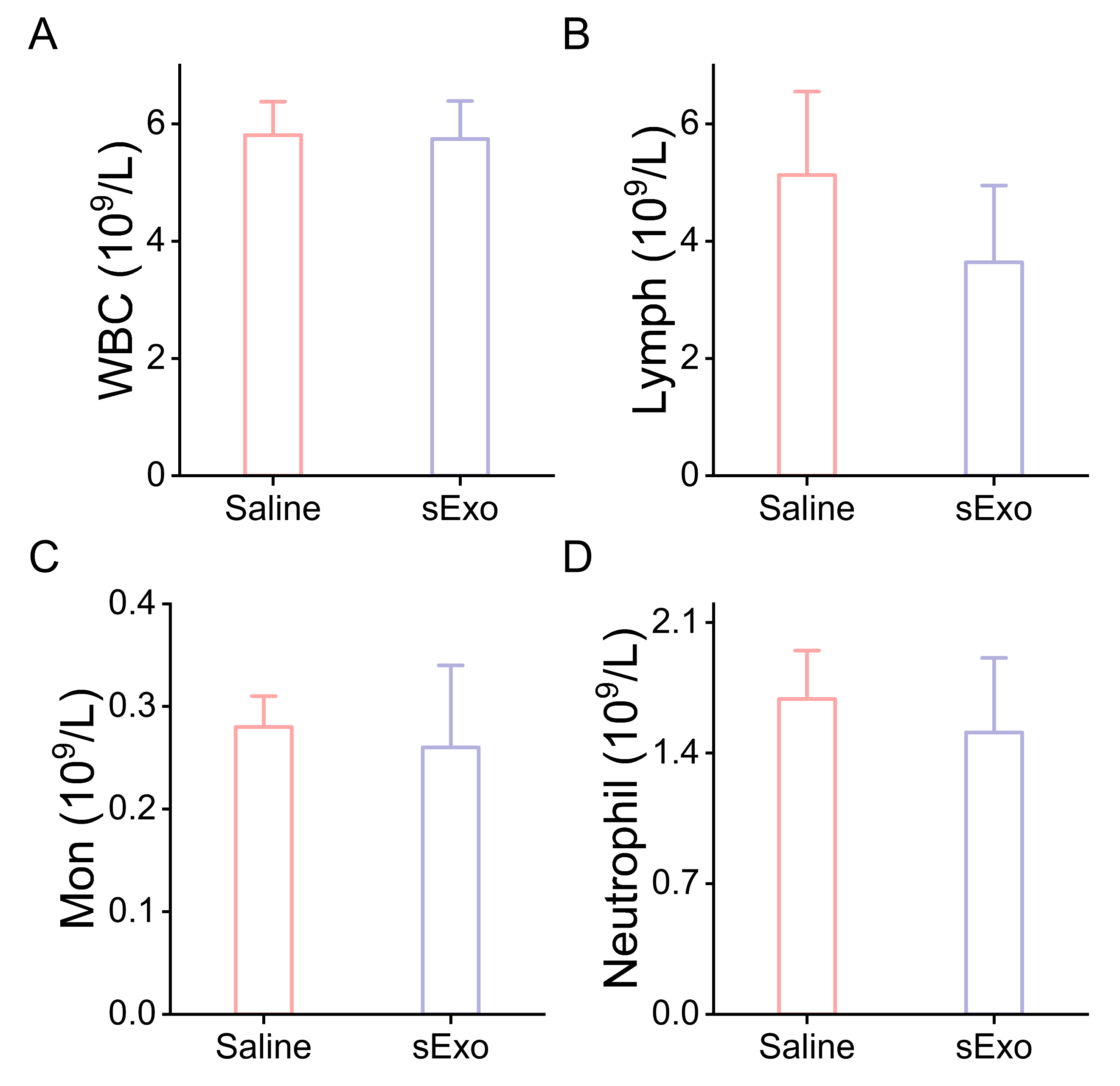


**Supplementary** Fig. S10. The contents of white blood cells (WBCs). **(A)**, lymphocytes **(B)**, monocytes **(C)**, and neutrophils **(D)** in mouse blood (n = 3).

*

*

**Supplementary Fig. S11.** H&E staining of the brain of the mice.





**Supplementary** Fig. S12. **(A)** Laser speckle imaging showing blood flow dynamics in the vasculature. (B) Quantitative rCBF analysis. (C) ELISA detection of CXCL4 and TXB2 levels.


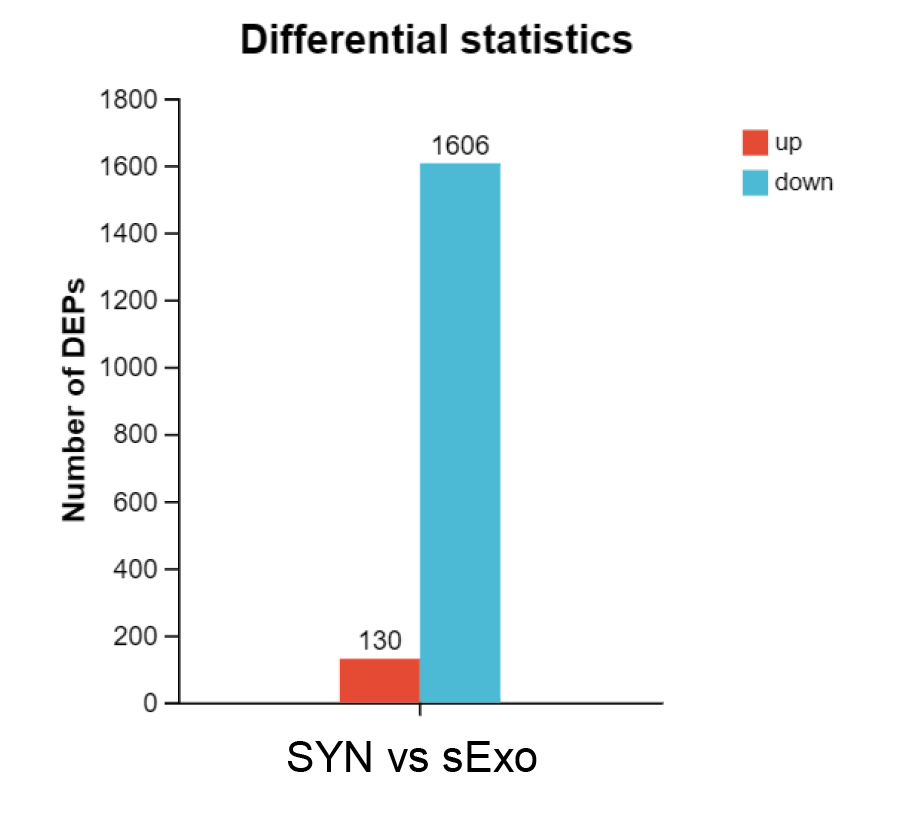


**Supplementary** Fig. S13. Differences in protein expression between SYN and sExo.


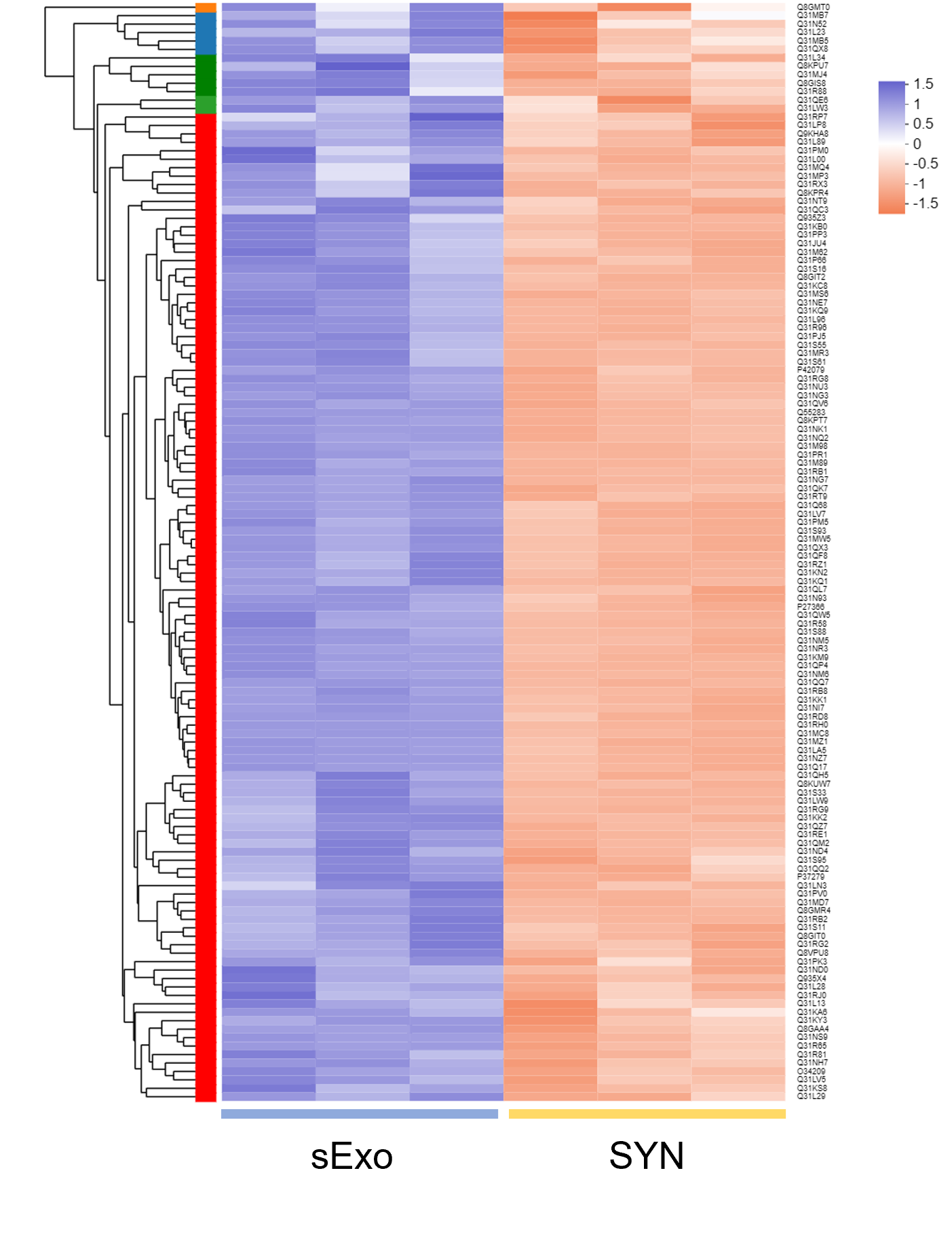


**Supplementary** Fig. S14. The heatmap shows sExo compared with SYN-upregulated proteins.


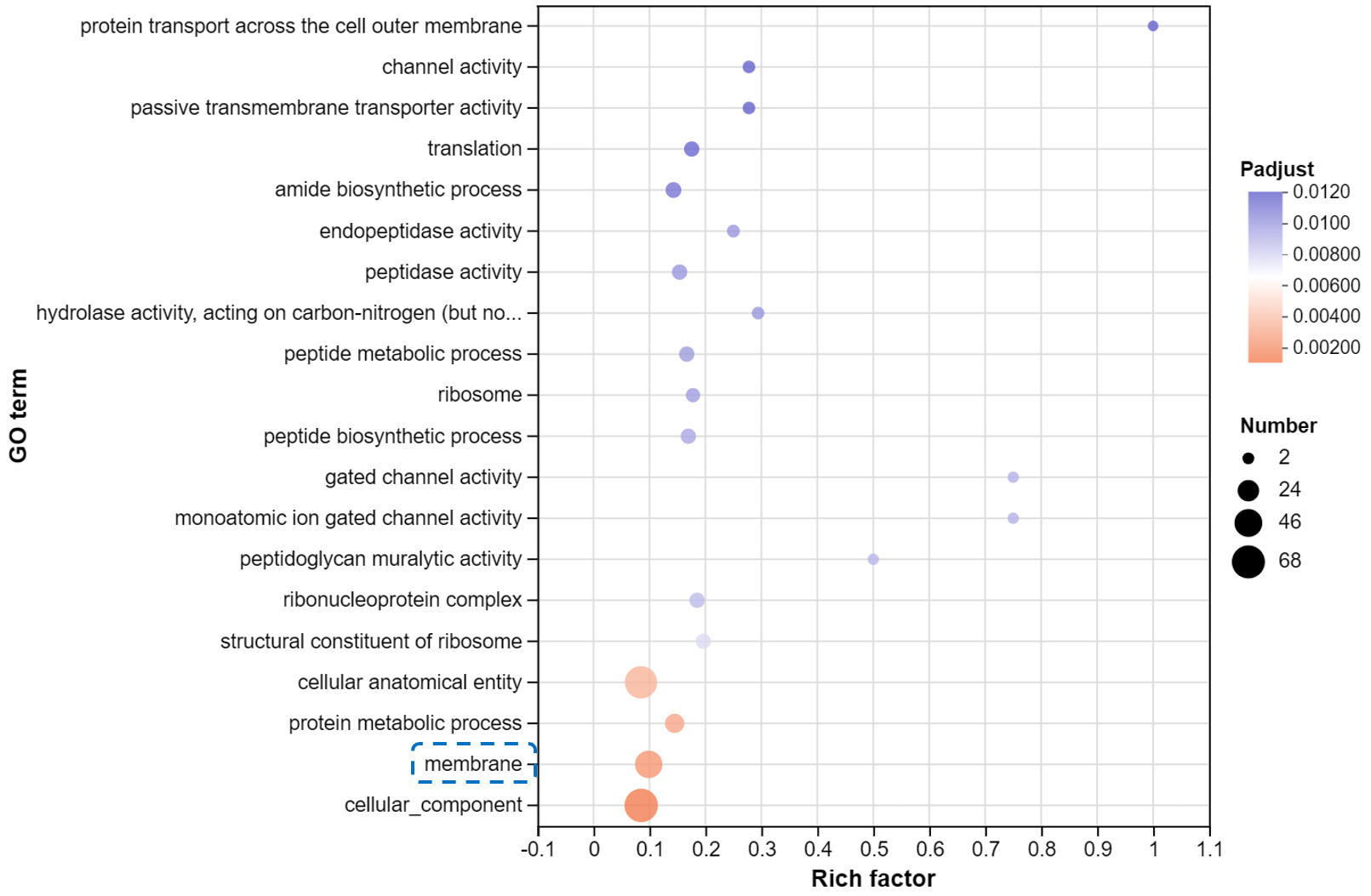


**Supplementary** Fig. S15. GO enrichment analysis of upregulated proteins in the sExo group.





**Supplementary** Fig. S16. (**A**) Three-dimensional binding model between integrin α_4_β_1_ (green) and Q31N76 (magenta) (left), and schematic diagram of interface residue interactions (right). **(B)** Three-dimensional binding model between ICAM-1 (green) and Q31IA4 (magenta) (left), and schematic diagram of interface residue interactions (right). **(C)** Three-dimensional binding model between P-gp (green) and Q31N76 (magenta) (left), and schematic diagram of interface residue interactions (right).





**Supplementary** Fig. S17. Inhibitor-based analysis of endocytic pathways involved in sExo transcytosis across the BBB.


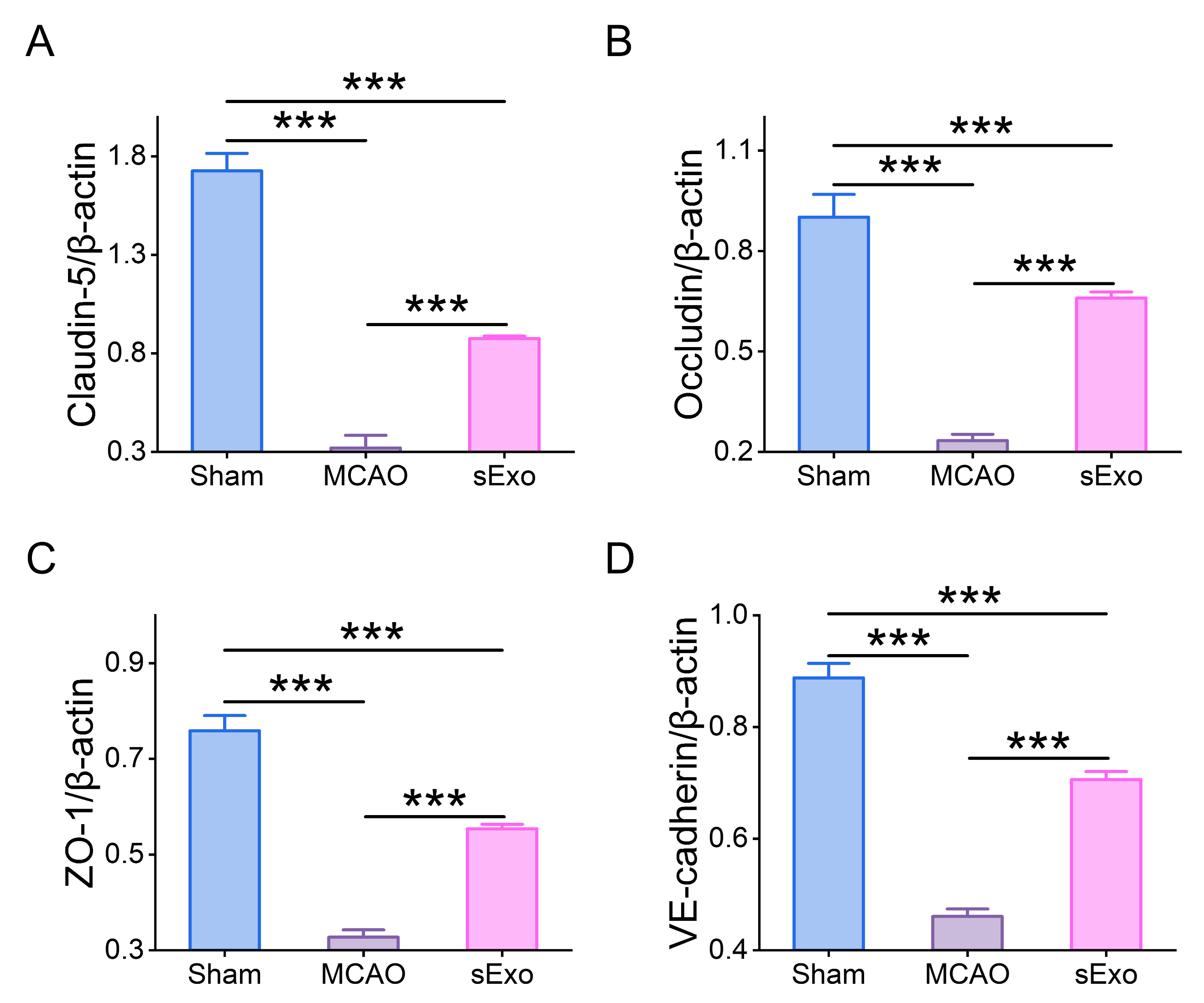


**Supplementary** Fig. S18. Protein expression levels of **(A)** Claudin-5, **(B)** Occludin, **(C)** ZO-1 and **(D)** VE-cadherin in each group (n = 3). One-way ANOVA was used to calculate *P* values (**P* < 0.05, ***P* < 0.01, ****P* < 0.001, ns, not significant).


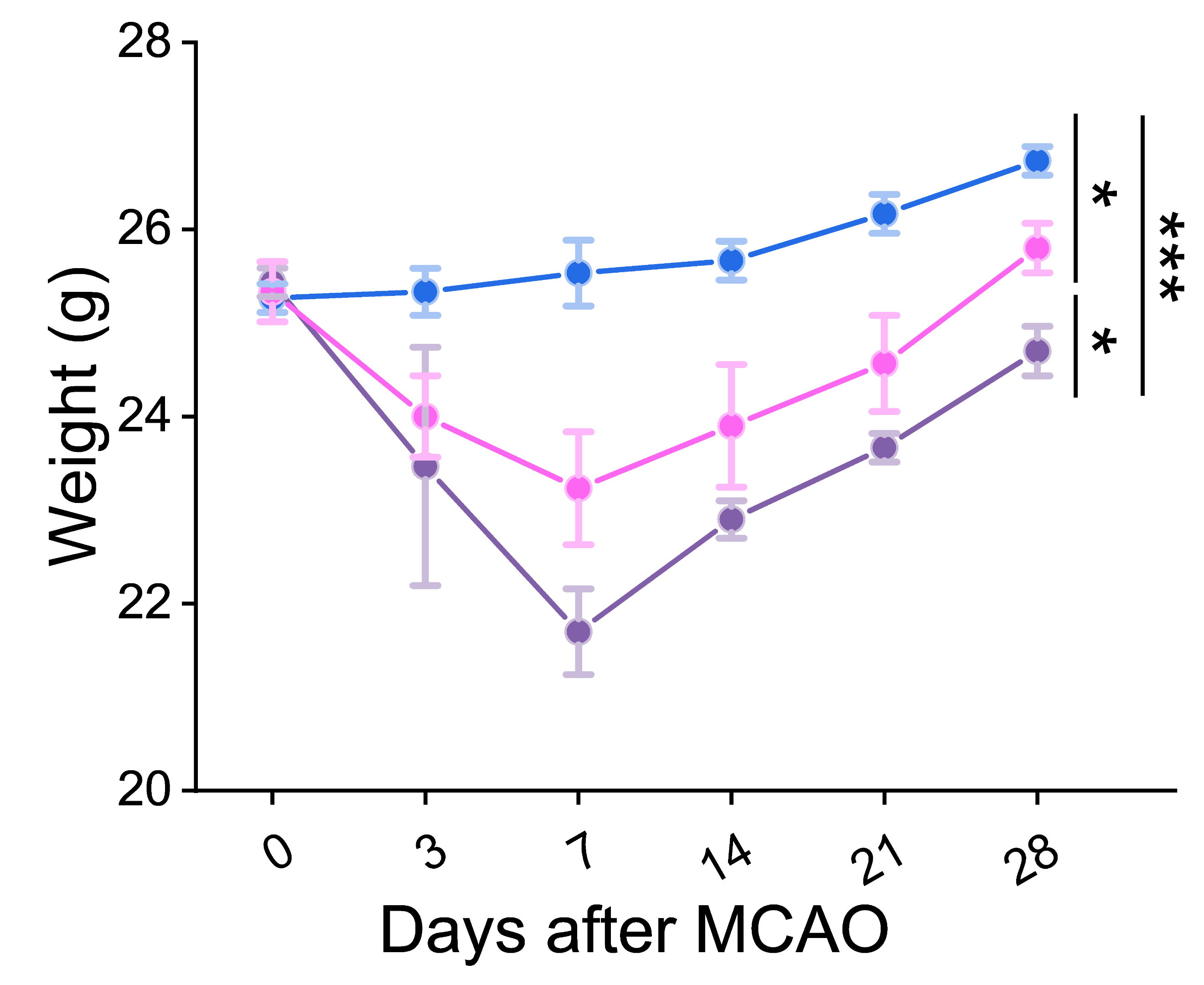


**Supplementary** Fig. S19. Changes in the weights of the mice in the different treatment groups (n =4). Two-way ANOVA was used to calculate *P* values (**P* < 0.05, ***P* < 0.01, ****P* < 0.001, ns, not significant).


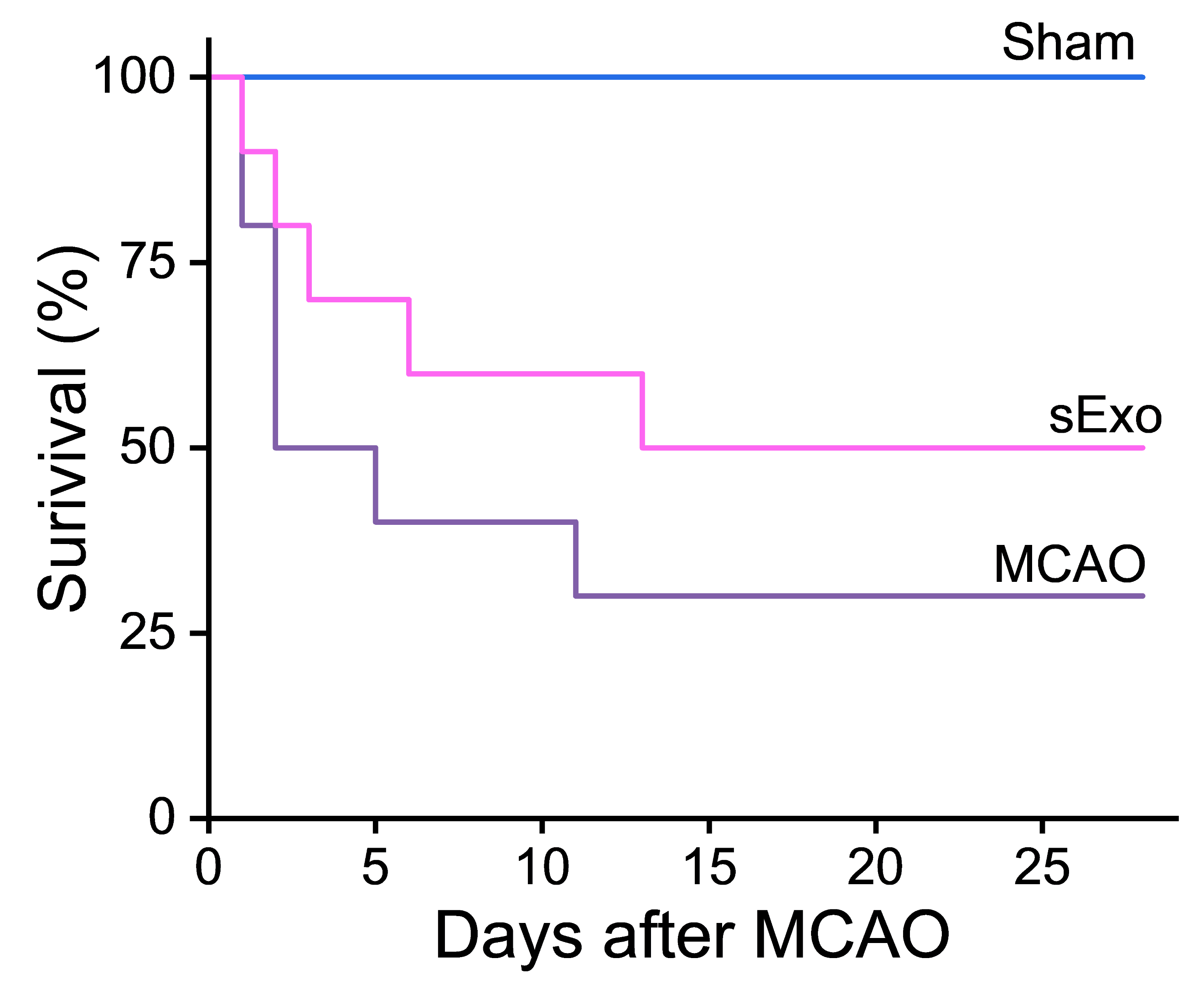


**Supplementary** Fig. S20. Survival rates of the differently treated mice 28 days after tMCAO (n = 10).





**Supplementary** Fig. S21. **(A)** Quantitative analysis of DHE staining. n=4. **(B)** ROS levels in mouse brains were measured *via* ELISA. (n=4).One-way ANOVA was used to calculate *P* values (**P* < 0.05, ***P* < 0.01, ****P*< 0.001, ns, not significant).


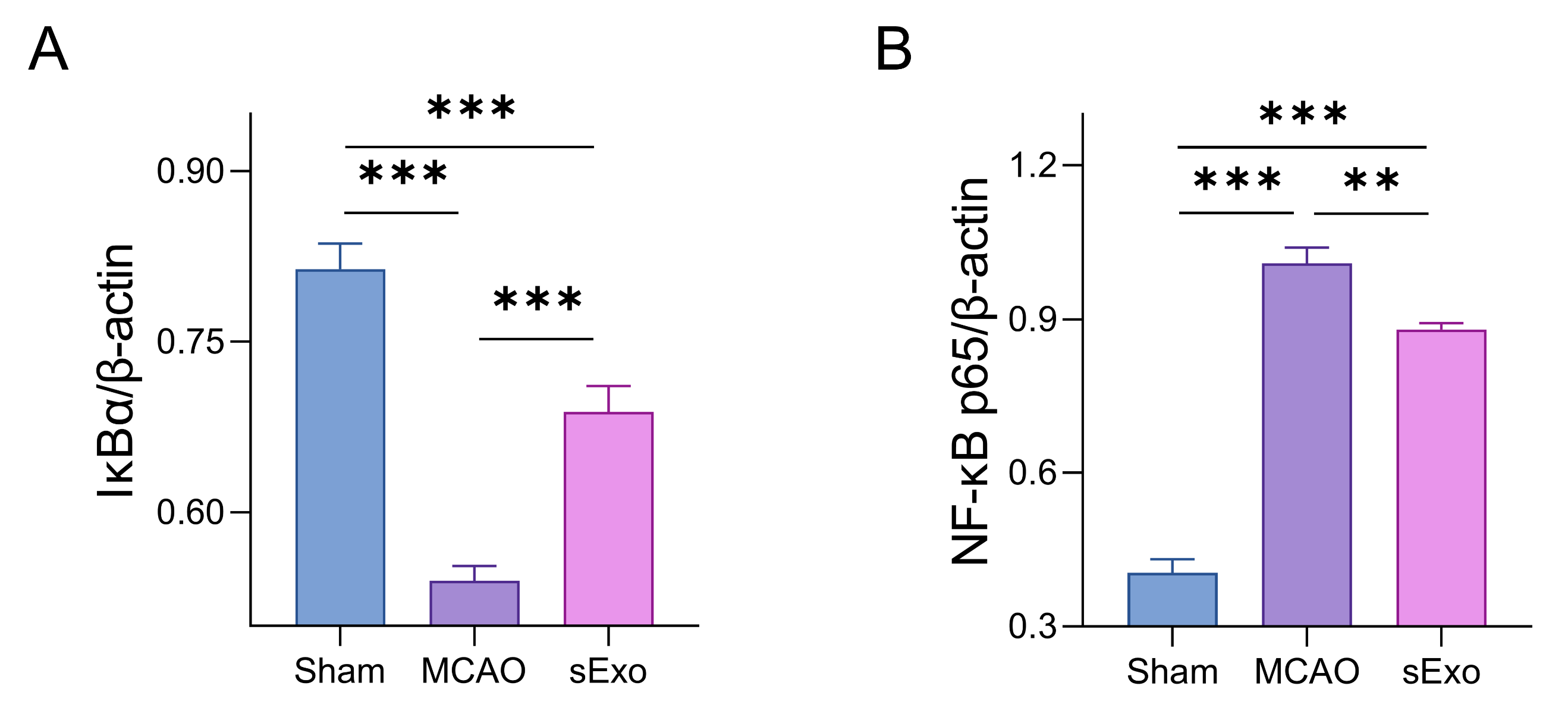


**Supplementary** Fig. S22. Protein expression levels of **(A)** IκBα and (B) NF-κB p65 in each group (n = 3). One-way ANOVA was used to calculate *P* values (**P* < 0.05, ***P* < 0.01, ****P* < 0.001, ns, not significant).


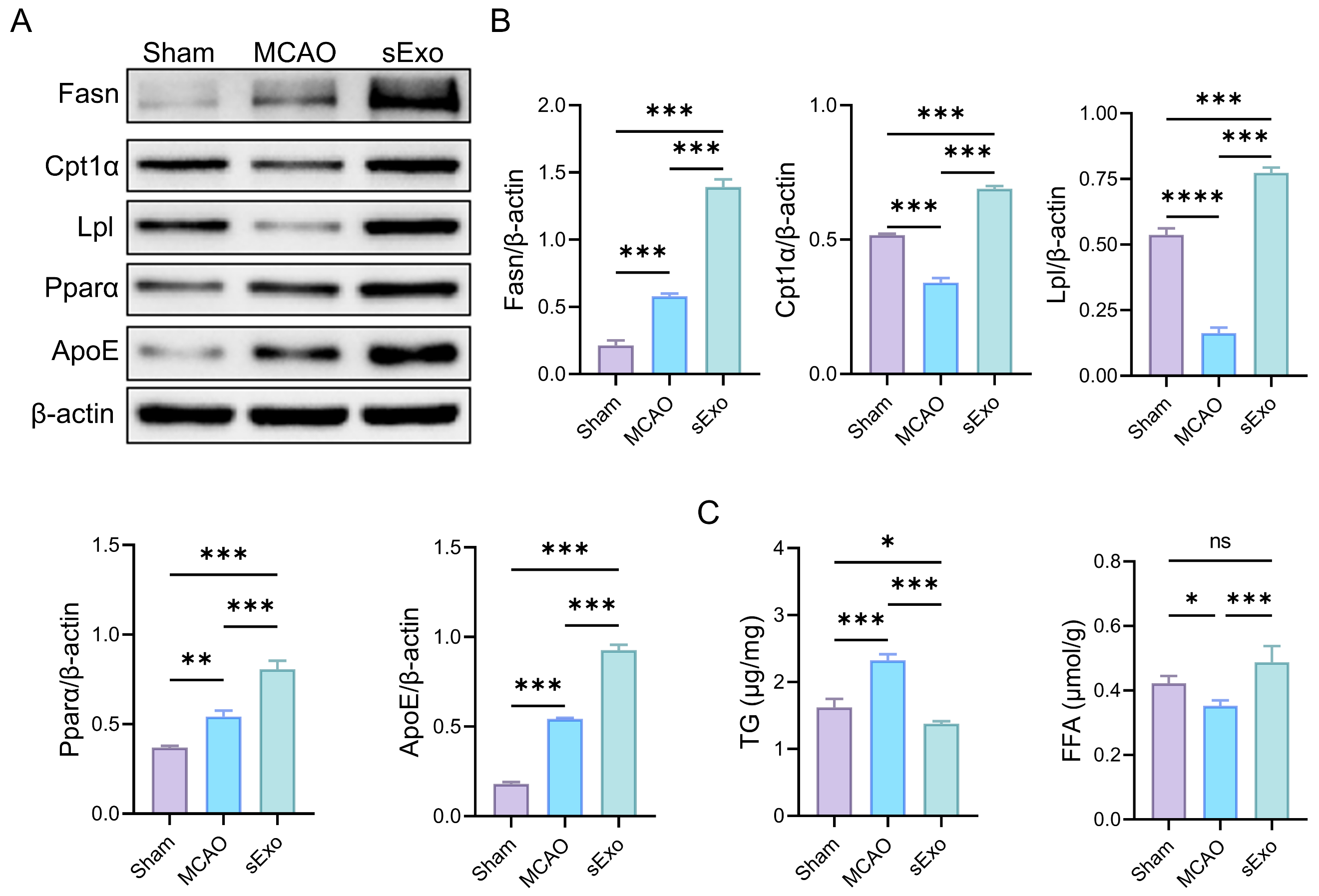


**Supplementary** Fig. S23. (A) WB analysis of Fasn, Cpt1α, Lpl, Pparα and ApoE in the ischemic brains of different mice. (B) Protein expression levels were measured in each group (n = 3). (C) ELISA detection of TG and FFA levels. One-way ANOVA was used to calculate *P* values (**P* < 0.05, ***P* < 0.01, ****P* < 0.001, ns, not significant).
